# *Akkermansia muciniphila* drives viscero-visceral crosstalk via 5-HT_3a_R-mediated sensitization of dichotomizing gut–bladder neurons

**DOI:** 10.1038/s12276-026-01720-4

**Published:** 2026-05-08

**Authors:** Qi Sun, Yubo Gao, Jun Zheng, Rulin Liao, Haotian Jiang, Zhangrui Zhu, Ming Xie, Yao Yu, Yuexuan Zhu, Weijia Li, Wentai Shangguan, Leqian Li, Xinhang Shi, Qishen Yang, Jiao Zeng, Zongwei Wang, Jie Zhao, Bisheng Cheng, Peng Wu

**Affiliations:** 1 Department of Urology, Nanfang Hospital, Southern Medical University, Guangzhou, China; 2 State Key Laboratory of Organ Failure Research, Key Laboratory of Mental Health of the Ministry of Education, Guangdong-Hong Kong-Macao Greater Bay Area Center for Brain Science and Brain-Inspired Intelligence, Guangdong-Hong Kong Joint Laboratory for Psychiatric Disorders, Guangdong Provincial Key Laboratory of Psychiatric Disorders, Guangdong Basic Research Center of Excellence for Integrated Traditional and Western Medicine for Qingzhi Diseases, Department of Neurobiology, School of Basic Medical Sciences, Southern Medical University, Guangzhou, China; 3 Department of Surgery, Division of Urology, Beth Israel Deaconess Medical Center, Harvard Medical School, Boston, MA USA; 4 NMPA Key Laboratory for Research and Evaluation of Drug Metabolism, Guangdong Provincial Key Laboratory of New Drug Screening, School of Pharmaceutical Sciences, Southern Medical University, Guangzhou, China; 5 Department of Clinical Oncology, Li Ka Shing Faculty of Medicine, The University of Hong Kong, Hong Kong SAR, China; Department of Clinical Oncology, The University of Hong Kong-Shenzhen Hospital, Shenzhen, China

**Keywords:** Experimental models of disease, Bladder disease, Enteric nervous system

## Abstract

The comorbidity of overactive bladder (OAB) and irritable bowel syndrome (IBS) presents a major clinical challenge, with the underlying neural and microbial mechanisms of the gut–bladder axis poorly understood. Here we aimed to delineate the complete causal pathway from a specific gut microorganism to bladder dysfunction and validate it as a therapeutic target. We combined analysis of human OAB–IBS cohorts with a postinflammatory mouse model, integrating retrograde neuronal tracing, multiomics (16S rDNA and metabolomics), fecal microbiota transplantation, urodynamics, dorsal root ganglion (DRG) electrophysiology and pharmacological and/or surgical interventions. We first confirmed a direct anatomical link, identifying dichotomized DRG neurons co-innervating the colon and bladder. Patients with OAB–IBS and mice exhibited a shared gut dysbiosis characterized by *Akkermansia muciniphila* enrichment. This comorbidity occurred in the absence of local bladder inflammation or urinary colonization with *A. muciniphila*, confirming a functional, noninfectious mechanism. Fecal microbiota transplantation of *A. muciniphila* or patient microbiota causally exacerbated visceral hypersensitivity, the OAB phenotype and DRG hyperexcitability. Mechanistically, *A. muciniphila* enrichment shunted host tryptophan metabolism toward the serotonin (5-HT) pathway. The resulting excess 5-HT acted on specifically upregulated colonic 5-HT_3a_ receptors to drive neuronal sensitization. Crucially, pharmacological blockade of the colonic 5-HT_3a_ receptor or surgical severing of the mesenteric nerves reversed the bladder dysfunction and visceral hypersensitivity. Our findings delineate a novel pathway wherein *A. muciniphila* drives functional gut–bladder comorbidity by promoting a gut-derived serotonergic signal that sensitizes shared afferent neurons, establishing the gut-specific 5-HT_3a_ receptor as a key, druggable therapeutic target.

## Gut-microorganism links to overactive bladder dysfunction revealed

Overactive bladder (OAB) and irritable bowel syndrome (IBS) often co-occur, affecting many adults and complicating treatment owing to unclear mechanisms. This study explores the gut–bladder connection, focusing on the role of the gut bacterium *Akkermansia muciniphila*. Researchers hypothesized that this bacterium influences OAB–IBS comorbidity by altering tryptophan metabolism, increasing serotonin production and activating sensory neurons. Using a mouse model, they demonstrated that *A. muciniphila* enrichment leads to increased serotonin levels and heightened neuron excitability, causing bladder dysfunction without local inflammation. The study found that blocking the serotonin receptor 5-HT3a or severing gut nerves alleviated symptoms, highlighting a neural pathway for gut–bladder communication. These findings suggest potential therapeutic targets, such as the 5-HT3a receptor, for treating OAB–IBS comorbidity, offering new insights into managing these disorders. Future research could explore microbiota modulation as a treatment strategy.

This summary was initially drafted using artificial intelligence, then revised and fact-checked by the author.

## Introduction

Overactive bladder (OAB) is a prevalent and debilitating functional disorder of the lower urinary tract, characterized by urinary urgency with or without incontinence, often accompanied by frequency and nocturia^1^. Affecting approximately 16–17% of the adult population, OAB substantially impairs quality of life and imposes a considerrable economic burden^2,3^. Current treatments are often limited in efficacy, largely owing to an incomplete understanding of the underlying pathophysiology in many patients, underscoring the urgent need for new mechanistic insights and therapeutic targets^4^. The current prevailing etiological theories for OAB include myogenic, urotheliogenic, supraspinal and urethrogenic hypotheses^5^. Metabolic syndrome, pelvic organ crosstalk, autonomic dysfunction and urinary microbial disorder are pathophysiological factors of OAB^5,6^. Among them, pelvic organ crosstalk between the bladder and the colon is an important pathogenesis subordinate to myogenic theory^5,7^.

A major clinical challenge is the frequent and well-documented comorbidity of OAB with functional bowel disorders, such as irritable bowel syndrome (IBS)^8,9^. Patients with OAB frequently report symptoms of visceral hypersensitivity (VH), including abdominal pain and bloating, and these symptoms are often considered when evaluating therapeutic outcomes^10^. Primary afferent neurons from the colon and bladder travel via the hypogastric (T10–L2) and pelvic (L6–S2) nerves and converge in the thoracolumbar (TL) and lumbosacral (LS) dorsal root ganglia (DRGs)^11,12^. These neurons, which include a key subpopulation of dichotomized neurons that innervate both organs, project their peripheral sensory terminals into the gut and bladder wall^13^. This shared circuitry provides a direct route for pathological signals from the gut to sensitize bladder afferent pathways, offering a compelling explanation for the co-occurrence of VH and bladder dysfunction^14^.

Recent advances have highlighted the gut microbiota as a master regulator of host physiology, profoundly influencing visceral sensitivity through the ‘gut–brain axis’^15,16^. This has led to the emerging concept of a ‘gut–bladder axis’, yet the mechanisms, particularly those involving neural regulation, remain largely unexplored^17^. The gut microbiota is known to produce a vast array of metabolites that can modulate host neurophysiology. A critical example is the metabolism of tryptophan, the precursor to serotonin (5-HT). Gut microorganisms can influence host tryptophan availability and regulate host 5-HT synthesis by intestinal cells, thereby impacting DRG neuron excitability and visceral pain^18^. However, whether a specific dysbiotic state can leverage the tryptophan–serotonin pathway to mediate gut–bladder crosstalk is unknown.

Here, we hypothesized that a specific gut microbial signature, characterized by the enrichment of *Akkermansia muciniphila*, drives OAB–IBS comorbidity by regulating host tryptophan metabolism. We proposed that this regulation shunts tryptophan toward the serotonin pathway, and the resulting excess gut-tropic 5-HT acts on specific receptors located on the peripheral terminals of these shared DRG neurons, inducing a state of hyperexcitability that manifests as bladder dysfunction. To test this, we first established a postinflammatory mouse model that recapitulates the human comorbidity and identified parallel microbial changes. We then used microbiota transplantation and in vitro assays to establish causality and dissected the complete molecular cascade from microbial metabolism to the specific 5-HT_3a_ receptor. Our findings delineate this novel pathway in its entirety and validate it as a viable therapeutic target, providing a new framework for understanding and treating gut–bladder disorders.

## Material and methods

### Patient recruitment and sample collection

Between January 2023 and April 2024, a total of 40 eligible individuals were identified from the outpatient clinic, including 20 patients with OAB–IBS and 20 asymptomatic controls, all of whom were female. Patients with OAB–IBS fulfilling the following criteria were considered eligible for this study: (1) female, 18 years of age or older; (2) presented with classic OAB symptoms defined by the International Continence Society; (3) Overactive Bladder Symptom Score (OABSS) total score ≥3 points and question 3 (urgency) score ≥2 points; (4) meeting the Rome IV criteria, including recurrent abdominal pain on average at least 1 day per week in the last 3 months; and (5) IBS Symptom Severity Scale (IBS-SSS) over 75 points at baseline. Participants were excluded if they met one or more of the following exclusion criteria: (1) urinary tract infection (based on the examination of urinary sediments), (2) presence of an indwelling catheter, (3) antibiotic exposure within the last 30 days, (4) chemical cystitis and cyclophosphamide cystitis, (5) medical history of inflammatory bowel disease, (6) history of gastrointestinal surgery and (7) pregnancy or breastfeeding. The inclusion and exclusion criteria were consistent for the control subjects, except for the absence of OAB or IBS symptoms. The scores of the IBS-SSS, OABSS and Visceral Sensitivity Index (VSI) were used to comprehensively evaluate the gut and bladder symptoms as well as visceral sensitivity in patients. Higher scores indicate a more severe condition in patients. Then, the feces of the enrolled subjects were collected into 15 ml sterile Epperdorf tubes and stored in a −80 °C freezer until use. The study protocol was approved by the Institutional Review Board of Nanfang Hospital (reference no. NFEC-2020-123). Written informed consent was provided by all participants before the collection of the samples.

### Animals

Female C57BL/6J mice (6–8 weeks old) were sourced from the Animal Center of Southern Medical University (Guangzhou, China). All C57BL/6J mice were housed in a specific pathogen-free environment with free access to food and water in a temperature-controlled room (22 ± 1 °C) with a relatively constant humidity of 45% ± 10% under a 12-h light–dark cycle. All experiments were conducted by the National Standards for the Care and Use of Laboratory Animals and were approved by the Institutional Animal Care and Use Committee (IACUC) of Nanfang Hospital, Southern Medical University (approval no. IACUC-LAC-20240401-013).

### TNBS-induced colitis model

Female C57BL/6 mice were anesthetized with an intraperitoneal injection of sodium pentobarbital (40 mg/kg) and administered with a single dose of 2,4,6-trinitrobenzenesulfonic acid (TNBS) (2.5 mg/mice; Sigma-Aldrich) in 50% ethanol via intrarectal injection. After administration of TNBS, mice were held in an inverted position for 30–60 s to ensure proper distribution of TNBS within the colon. The control group received 50% ethanol without TNBS. Colonic inflammation subsided within 7 days and transitioned to a chronic inflammatory state by day 28, at which point these mice developed chronic colonic afferent hypersensitivity^19^. Mice were assessed daily for changes in body weight, fecal consistency and gross bleeding, with scores assigned to calculate a composite disease activity index (DAI)^20^. All mice were killed on day 28, followed by a measurement of the colon length.

### Study design

First was the establishment of a model of gut–bladder axis disruption. Mice were randomly assigned to two groups: (1) control and (2) TNBS. The gastrointestinal function of the mice was assessed on day 3 and day 28, and mechanical sensitivity was evaluated every 7 days. The bladder function of the mice was assessed on day 14 and day 28. Following being killed on day 28, the DRGs from the L6 and S1 segments were collected for patch-clamp recording and cell culture^19,21^.

Second was the effect of gut microbiota on the gut–bladder axis. Mice were randomly assigned to four groups: (1) control, (2) TNBS, (3) Akk-FMT (where FMT is fecal microbiota transplantation) and (4) VH-FMT. Apart from the control group, the remaining three groups received intrarectal TNBS injections. Before the FMT experiment, recipient mice were administered oral antibiotics (vancomycin 100 mg/kg, neomycin sulfate 200 mg/kg, metronidazole 200 mg/kg, ampicillin 200 mg/kg) once daily for 1 week to deplete the gut microbiota. In the Akk-FMT group, before drug administration, *A. muciniphila* (American Type Culture Collection BAA835) frozen pellets were thawed and resuspended in anaerobic PBS to a concentration of 1.5 × 10^9^/ml. Mice were orally administered a suspension of *A. muciniphila* in brain heart infusion (BHI) twice weekly for 3 weeks^22^. In the VH-FMT group, feces collected from donors (patients with OAB–IBS) was placed in sterile centrifuge tubes, resuspended in PBS at a concentration of 125 mg/ml and subsequently administered via gavage with the same frequency as the Akk-FMT group. The control and TNBS mice received PBS gavage at an identical schedule.

Third was the effect of gut nerve blockade on the gut–bladder axis. Mice were randomly assigned to three groups: (1) TNBS, (2) ondansetron and (3) surgery. Each group received intrarectal TNBS injections. The nerve blockade procedures for both groups were carried out on day 21 following the TNBS injection. In the drug ondansetron group, ondansetron was administered via mesenteric artery injection to specifically block intestinal nerve conduction^23^. In the surgery group, the mesentery of the large intestine was bluntly dissected up to the ileocecal junction using forceps^24^.

### Imaging of in vivo intestinal inflammation

Mice were induced with anesthesia by inhalation of 3% isoflurane and maintained with 1–1.5% isoflurane. They were then injected with 100 µl of a 20 mmol/l L-012 (FUJIFILM Wako Pure Chemical Corporation) solution^25^. A total of 1 min later, bioluminescent images were captured using the IVIS Spectrum CT System. For postacquisition analysis, the bioluminescent signals from a standardized region of interest (ROI) defined over the abdomen were quantified using Living Image software.

### Assessment of visceral sensitivity

Visceral sensitivity in mice was evaluated by changes in abdominal electromyography (EMG) and scores of the abdominal withdrawal reflex (AWR) in response to colorectal distension (CRD). The amplitude of EMG was measured using the BL-420N multichannel physiological signal acquisition and processing system (Chengdu Techman Software). In the CRD procedure, distension pressures of 20, 40 and 60 mmHg were used, each lasting for 20 s and applied at intervals of 4 min. To ensure the accuracy of the experiment, mice were fasted for 12 h before visceral sensitivity testing but had free access to water. After anesthesia induced by intraperitoneal injection of sodium pentobarbital (40 mg/kg), a balloon catheter coated with paraffin oil was inserted into the rectum of the mice, with the tail end of the balloon approximately 3 cm from the anus. The end of the catheter connected to the balloon was securely taped to the tail to prevent slippage. The mice were allowed to recover and acclimate for 1 h before undergoing CRD. During the distension phase, EMG activity was recorded under different pressure conditions and the AWR score was assessed simultaneously^26,27^. The AWR scoring criteria were as follows: 0, no behavioral response to colorectal distension (CRD); 1, the mouse becomes immobile during the CRD and occasionally clinches the head at the onset of the stimulus; 2, a mild contraction in the abdominal muscles is observed, but the mouse does not lift its abdomen off the platform; 3, a strong contraction of the abdominal muscles is observed and the mouse lifts its abdomen off the platform; 4, a severe contraction of the abdominal muscles is manifested by body arching and the mouse lifts its pelvic structures off the platform^28^. The AWR measurements were conducted by a blinded observer who assigned AWR scores on the basis of the responses of the mice to CRD (20, 40 and 60 mmHg).

### Von Frey test

Mice were acclimated for at least 30 min in an acrylic cube on a wire mesh floor. A series of ten von Frey filaments (BIOVF-M, Bioseb) with bending forces of 0.008, 0.02, 0.07, 0.16, 0.4, 0.6, 1, 1.4, 2 and 4 g were selected. Licking or scratching the stimulated area, rearing or rapidly jumping up were all considered positive behavioral responses^29^. Measurements were repeated three times at 5-min intervals, and the average value was taken as the mechanical withdrawal threshold at a specific time point^30^.

### Cystometry

Mice were induced with anesthesia via inhalation of 3–4% isoflurane and subsequently maintained with 1–1.5% isoflurane and then a PE-50 catheter was advanced retrograde into the bladder and connected to a pressure transducer (BL-420F, Taimeng Technology) in line with an infusion pump. Sterile normal saline was infused at 2.4–3.0 ml/h and intravesical pressure was continuously recorded with BL New Century 2.1 software (Taimeng Technology). After voiding cycles stabilized (typically three to four cycles), an additional 15 min was recorded for quantitative analysis. The recorded cystometrography parameters included intravesical pressure (baseline, threshold and peak micturition pressure), bladder volume, the frequency of voiding contractions (VCs) and non-VCs and intercontraction interval.

### Neuronal retrograde tracing

Retrograde tracer injection was performed 7 days before collection DRGs. Mice were anesthetized with isoflurane (3–4%), and the bladder and distal colon were exposed via a lower abdominal midline incision. To label bladder-innervating neurons, the bladder wall/trigone was injected with DiI (2 mg/100 µl in dimethylsulfoxide; 10 µl total distributed into three to four sites). To label colon-innervating neurons, the distal colon wall was injected with Fast Blue (FB; 1 mg/100 µl in sterile saline; 10 µl total distributed into three to four sites). To prevent tracer cross-contamination, any leakage from injection sites was immediately removed using a cotton-tipped applicator, and the injection sites were washed with sterile saline. The peritoneal cavity was rinsed with sterile saline before suturing the muscle and skin layers separately.

### Isolation and culture of DRG neurons

DRGs were located within the intervertebral foramina adjacent to the spinal cord. Therefore, laminectomy was conducted before isolating the DRG (L6 and S1)^31^. The collected DRGs were placed into 1.5-ml centrifuge tubes, into which the extraction medium (MEM-ALPHA medium + 2% fetal bovine serum (FBS)) was added. After extraction, the extraction medium in the 1.5-ml centrifuge tube was slowly aspirated and discarded, followed by the addition of 1 ml of the mixed digestive enzyme working solution (Collagenase I + Dispase, 5 mg/ml). The samples were then digested in a water bath at 37 °C for 60–70 min, with inversion and mixing every 10 min. Subsequently, the mixed digestive enzyme was aspirated and discarded, and 1 ml complete culture medium (MEM-ALPHA medium + 10% FBS + 1% penicillin–streptomycin) was added. The mixture was filtered through a 200-mesh cell strainer to remove impurities and then centrifuged at 1000 rpm for 8 min. After centrifugation, the supernatant was discarded and the pellet was resuspended in 1 ml complete culture medium. The cells were counted and then plated into 35-mm culture dishes, with an additional 1 ml complete culture medium added. The dishes were then placed into a temperature-controlled incubator (37 °C, 5% CO_2_) for 8–12 h. After this period, the medium was replaced with fresh medium (MEM-ALPHA medium + 5% FBS + 1% penicillin–streptomycin + 1% 5-Fluoro) to maintain cell growth and, thereafter, half of the medium was changed every 2 days.

### Whole-cell patch-clamp recordings of DRG neurons

Whole-cell patch-clamp recordings were performed in primary-cultured DRG neurons with a MultiClamp 700B amplifier and a Digidata 1550B digitizer (Axon Instruments)^32^. Borosilicate glass pipettes with a resistance of 3–6 MΩ were filled with an internal solution containing (in mM): potassium gluconate 126, NaCl 10, MgCl_2_ 1, EGTA 10, Na-ATP 2 and Mg-GTP 0.1, adjusted to pH 7.3 with KOH. The external solution contained (in mM): NaCl 140, KCl 5, CaCl_2_ 2, MgCl_2_ 1, HEPES 10 and glucose 10, adjusted to pH 7.4 with NaOH osmolarity to 300–310 mOsm^33^. In experiments using current clamp, action potentials (APs) were elicited through the application of a current pulse.

### Immunohistochemistry

After anesthesia, mice were perfused transcardially with PBS, followed by 4% paraformaldehyde. Following the perfusion, the DRG neurons from the L6 and S1 segments were collected and placed in 4% paraformaldehyde overnight, then transferred to 30% sucrose for tissue dehydration at 4 °C. DRG sections (10 μm) were subjected to immunofluorescence staining. The sections were blocked with 2% BSA at room temperature for 2 h, followed by incubation with primary antibodies overnight at 4 °C. The sections were then washed three times in PBS for 5 min each, followed by incubation with secondary antibodies at room temperature for 2 h, and then washed again. The stained sections were examined using a Nikon fluorescence microscope, and images were captured with a charge-coupled device spot camera. DRG cells were washed three times with PBS and fixed in 4% paraformaldehyde for 20 min. After treatment with 0.3% Triton X-100 (cat. no. 9002-93-1; Beijing Solarbio Science & Technology), for 10 min, the cells were blocked with 5% BSA for 1 h and then incubated with primary antibodies overnight at 4 °C. Subsequently, the cells were washed and incubated with secondary antibodies at room temperature for 60 min. Nuclei were stained with 4,6-diamidino-2-phenylindole (DAPI). The antibodies were used at the following dilutions: rabbit anti-TRPV-1 (1:1,000), goat anti-c-Fos (1:1,000) and rabbit anti-PGP9.5 (1:2,000) primary antibodies; fluorescein isothiocyanate; and cyanine3 (Cy3)-conjugated secondary antibodies (1:400)^32^. The fluorescence intensity was measured using National Institutes of Health ImageJ software.

### 16S rDNA gene sequencing

As previously described, microbial DNA was extracted from fecal samples^34^. PCR amplification of the V3–V4 region of 16S rDNA was performed using the following primer pairs on the Illumina Nova platform: 341F, 5′-CCTACGGGNGGCWGCAG-3′ and 806R, 5′-GGACTACHVGGGTATCTAAT-3′. Amplicons were purified using the AxyPrep DNA Gel Extraction Kit (cat. no. AP-GX-250; Axygen; Corning). Equimolar pooling of purified amplicons was performed and subjected to paired-end sequencing (PE250) on the Illumina platform according to the standard protocol. Alpha diversity was calculated in QIIME (version 1.9.1). The abundance statistics of each taxonomy were visualized using Krona (version 2.6). Principal coordinate analysis (PCoA) of weighted unifrac distances was generated in the R project Vegan package (version 2.5.3).

### Bacterial culture

The *A. muciniphila* (American Type Culture Collection BAA835) was cultured in BHI medium (Qingdao Hope Bio-Technology, HB5190) under anaerobic conditions at 37 °C^22^.

### Gene expression analysis

Total RNA was extracted from the colon and bladder using a total RNA isolation kit (Foregene), according to the manufacturer’s instructions. cDNA was obtained using the HiScript III RT SuperMix for Quantitative PCR (+gDNA wiper; Vazyme) with the following conditions: 50 °C for 15 min and 85 °C for 5 s. Quantitative PCR reactions were performed with an initial denaturation at 95 °C for 30 s, followed by 40 cycles of amplification at 95 °C for 10 s and 60 °C for 30 s. Melting curve analysis was then conducted at 95 °C for 1 min and 95 °C for 15 s using the ChamQ SYBR qPCR Master Mix (Vazyme Biotech) on a LightCycler480 (Roche Diagnostics). The relative quantification of target genes was calculated using the 2^−ΔΔCq^ method. Glyceraldehyde-3-phosphate dehydrogenase (GAPDH) was used as the reference gene. The sequences of all primers for the target genes are presented in Supplementary Table 2.

### Western blot analysis

Total protein was extracted from mouse colonic tissues by homogenization in RIPA Lysis and Extraction Buffer (Thermo Scientific) supplemented with a 1% protease and phosphatase inhibitor cocktail (CWBIO). The homogenates were briefly sonicated on ice and subsequently centrifuged at 12,000*g* for 10 min at 4 °C. The supernatant containing the total protein was collected, and the protein concentration was determined using a BCA Protein Quantification Kit (Thermo Scientific). For each sample, 20 μg of total protein was separated on a 10% SDS–polyacrylamide gel and transferred to a polyvinylidene difluoride membrane (Millipore). The membranes were blocked with 5% (w/v) BSA in Tris-buffered saline with Tween 20 for 2 h at room temperature. Subsequently, the membranes were incubated with a primary antibody against the 5-HT_3a_ receptor (Abcam, 1:5,000) overnight at 4 °C. After washing, membranes were incubated with the appropriate horseradish peroxidase-conjugated secondary antibody (Cell Signaling Technology, 1:5,000) for 1 h at room temperature. Immunoreactive bands were visualized using an enhanced chemiluminescence solution and captured with an imaging system (Santa Cruz Biotechnology). To ensure equal loading, membranes were stripped and reprobed with a primary antibody for GAPDH (Abcam, 1:2,000), followed by the corresponding secondary antibody. Band densitometry was performed using ImageJ software (National Institutes of Health), and the expression of the 5-HT_3a_ receptor was normalized to GAPDH.

### Targeted metabolomics analysis

Targeted metabolomics analysis of cells was performed using the Q300 Metabolite Assay Kit from Metabo-Profile Biotechnology, following a previously established protocol^35^. In brief, cell samples were collected on ice and subsequently lyophilized. For metabolite extraction, 150 μl precooled methanol-water (80:20, v/v) was added to the lyophilized cells, followed by ultrasonication-induced lysis. The lysate was then centrifuged at 13,000 rpm for 15 min at 4 °C. Next, 25-μl aliquots of the supernatant were transferred to a 96-well plate using a Biomek 4000 workstation (Beckman Coulter). Precooled methanol containing internal standards was automatically added to each well, and the mixture was vortexed for 5 min before centrifugation at 4000*g* for 30 min. Thereafter, 30 μl supernatant was derivatized with 20 μl freshly prepared derivatization reagents in a new 96-well plate. After 60 min of derivatization at 30 °C, the reaction was halted by adding 350 μl ice-cold 50% methanol solution and incubating the mixture at −20 °C for 20 min. Following another centrifugation at 4000*g* for 30 min at 4 °C, 135 μl supernatant was transferred to a clean 96-well plate containing 15 μl internal standards per well. The plate was sealed, and 5 μl supernatant was injected into the ultraperformance liquid chromatography coupled to tandem mass spectrometry (UPLC–MS/MS) system (ACQUITY UPLC-Xevo TQ-S, Waters) for analysis.

### Levels of tryptophan, kynurenine, 5-HT and 5-HIAA measurement

The levels of tryptophan (Abnova), kynurenine (Abnova), 5-HT (Abcam) and 5-HIAA (China) protein levels in mice colon were tested using commercial enzyme-linked immunosorbent assay (ELISA) kits according to the kit instructions.

### Levels of BDNF, NGF and CGRP measurement

The BDNF (Abnova), NGF (Abnova) and CGRP (Abnova) protein levels in DRGs were tested using commercial ELISA kits according to the kit instructions.

### Statistical analysis

The results are expressed as the mean ± standard error of mean (s.e.m.) and were calculated using GraphPad Prism software (version 8.0) and R software (version 4.1.1.). The difference between two groups was compared using the *t*-test or Mann–Whitney *U* test for continuous variables and using the chi-squared test for categorical variables. The differences among multiple groups were analyzed using one-way analysis of variance (ANOVA). To assess the relationships among all variables, pairwise correlations were evaluated using both Pearson and Spearman correlation coefficients. This approach allowed for the examination of both linear and monotonic associations between each pair of variables. In addition, odds ratios with corresponding 95% confidence intervals were calculated for categorical variables to further quantify the strength and significance of these associations. All *P* values were two-tailed, and the statistical significance level was set as 0.05.

## Results

### Clinical characteristics and symptom correlation in patients with OAB–IBS

A total of 20 patients with OAB and 20 asymptomatic controls were included in the study. Demographic characteristics and clinical information are presented in Supplementary Table 1. On the basis of the Rome IV criteria, the OAB–IBS patient cohort was classified into subtypes, consisting of IBS-mixed (IBS-M, *n* = 8), IBS-diarrhea (IBS-D, *n* = 6), IBS-constipation (IBS-C, *n* = 5) and IBS-unsubtyped (IBS-U, *n* = 1). There were no differences between the two groups in terms of age, body mass index (BMI) and history of hypertension or diabetes. To investigate the clinical relationship between bladder symptoms, bowel symptoms and visceral sensitivity within the patient cohort, we analyzed the correlations between the OABSS, the IBS Symptom Severity Score (IBS-SSS) and the VSI. As hypothesized, we found a strong and significant positive correlation between OABSS and both IBS-SSS (*r* = 0.5974, *P* = 0.0054) and VSI (*r* = 0.6501, *P* = 0.0019) (Supplementary Fig. 1). These clinical data confirm that in patients with OAB–IBS comorbidity, the severity of bladder symptoms is closely linked to the severity of both gut-related symptoms and VH^9,36,37^.

### TNBS-induced colitis establishes a model of sustained gut–bladder comorbidity

**Fig. 1:**
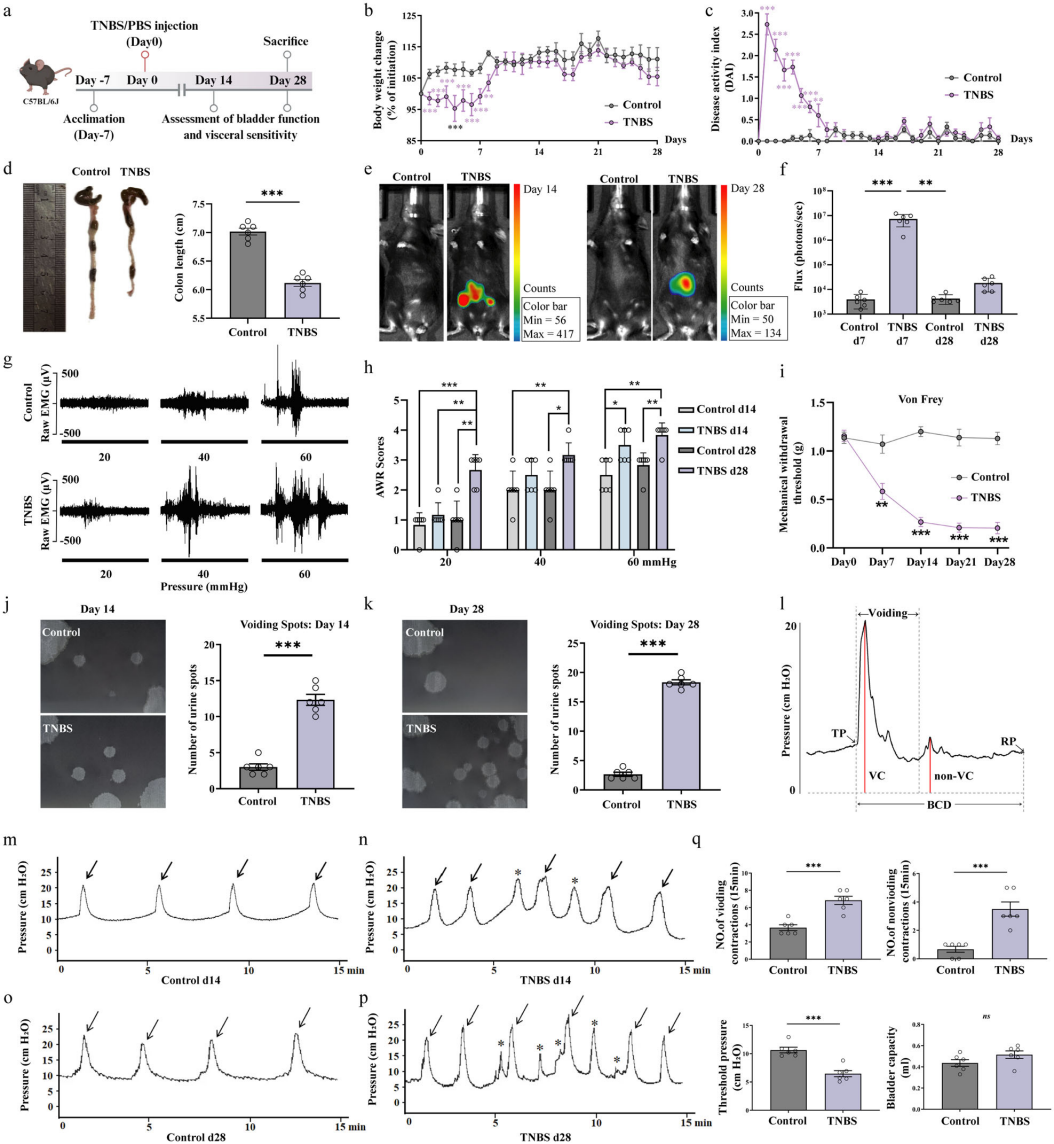
TNBS-induced colitis establishes a murine model of gut–bladder comorbidity featuring concurrent VH and OAB. **a** Schematic diagram illustrating the experimental design, timeline for TNBS induction and subsequent behavioral and physiological assessments. Colitis was induced by a single intrarectal administration of TNBS, with control mice receiving a vehicle. **b**, **c** TNBS-treated mice showed significant body weight loss (**b**) and increased DAI scores (**c**) during the first 7 days, and colon shortening (**d**) compared with controls. **e** Representative bioluminescence images of the control and the TNBS-induced group 14 days after injection (day 14) and 28 days after injection (day 28). **f** Flux values show the intestinal inflammation in mice of both the control and the TNBS-induced group. **g** Representative EMG traces of VMRs (20–60 mmHg, 20-s duration, 4-min intervals) in control and TNBS-treated mice. **h** AWR scores at varying degrees of colonic distension. **i** von Frey test. **j** Representative voiding-spot assay images and quantification of urine-spot numbers on day 14. **k** Representative voiding-spot assay images and quantification of urine-spot numbers on day 28. **l** A diagram illustrating the measurement of urodynamic parameters from a cystometry. RP, post-void resting pressure; BCD, bladder contraction duration. **m**–**p** Representative continuous cystometry records on day 14 and day 28 for both groups. **q** The urodynamic parameters evaluated included VCs (arrows), non-VCs (stars), TP and bladder capacity. Data are presented as mean ± s.e.m.. The statistical difference of various indicators between the two groups was evaluated using a two-tailed unpaired Student’s *t*-test, and a statistically significant difference was defined when *P* < 0.05. Statistical significance: *P < 0.05; **P < 0.01; ***P < 0.001. The schematic diagram in panel a was created by the authors using BioRender (https://biorender.com) in 2025. Figure created in BioRender.

To investigate the relationship between gut inflammation and bladder dysfunction, we first established a murine model of colitis via a single intrarectal administration of TNBS^36^ (Fig. 1a). Following the induction, TNBS-treated mice exhibited classic signs of acute colitis, including a significant loss of body weight and a corresponding increase in the DAI scores, with these systemic symptoms peaking and then resolving within the first 7 days (Fig. 1b, c). Morphological analysis revealed that TNBS-treated mice had markedly shorter colons compared with controls, a hallmark of inflammatory damage (Fig. 1d). Furthermore, in vivo bioluminescence imaging confirmed persistent inflammatory activity in the colonic region of TNBS mice on both day 14 and day 28, as indicated by significantly higher flux values (Fig. 1e, f). Next, we assessed whether this postinflammatory state was associated with lasting visceral and somatic hypersensitivity. On days 14 and 28, visceromotor responses (VMRs) to graded CRD were evaluated. As shown by representative EMG recordings and quantified AWR scores, TNBS-treated mice displayed markedly augmented responses to CRD compared with control mice (Fig. 1g, h). This VH was accompanied by referred somatic hypersensitivity, as the mechanical withdrawal threshold of the suprapubic region, determined by the von Frey test, was substantially lower in the TNBS group at these chronic time points (Fig. 1i).

To determine if this gut-centric pathology induced local inflammation in the bladder, we performed histological and molecular analyses. At both day 14 and day 28, the bladder of TNBS-treated mice showed no histopathological abnormalities via hematoxylin and eosin staining and no significant upregulation of key inflammatory markers (tumor-necrosis factor, interleukin (IL)-1β, IL-6), indicating an absence of cystitis (Supplementary Fig. 2a, b, e, f). This was in stark contrast to the colon, which displayed persistent low-grade inflammation at the same time points (Supplementary Fig. 2c, d, g, h). Having established that bladder dysfunction occurred in the absence of local inflammation, we proceeded to characterize the OAB phenotype. The voiding-spot assay revealed that TNBS mice produced a substantially greater number of urine spots on both day 14 and day 28, indicative of increased voiding frequency (Fig. 1j, k). To confirm this with gold-standard methodology, we performed continuous cystometry (Fig. 1l). The representative continuous cystometry records from TNBS mice on both day 14 and day 28 showed a clear increase in the frequency of both VCs (arrows) and non-VCs (stars) compared with controls (Fig. 1m–p). Quantitative analysis of the urodynamic parameters (Fig. 1q) demonstrated that TNBS-treated mice exhibited a significantly higher frequency of both voiding and non-VCs, coupled with a lower threshold pressure (TP) for inducing micturition. However, there was no significant difference in bladder capacity between the two groups. Taken together, these data demonstrate that a single episode of TNBS-induced colitis is sufficient to establish a robust and durable mouse model of gut–bladder comorbidity, characterized by sustained VH and a clear OAB phenotype that develops and persists without evidence of local bladder pathology.

### TNBS-induced colitis enhances the excitability of LS DRG neurons that co-innervate the colon and bladder

**Fig. 2:**
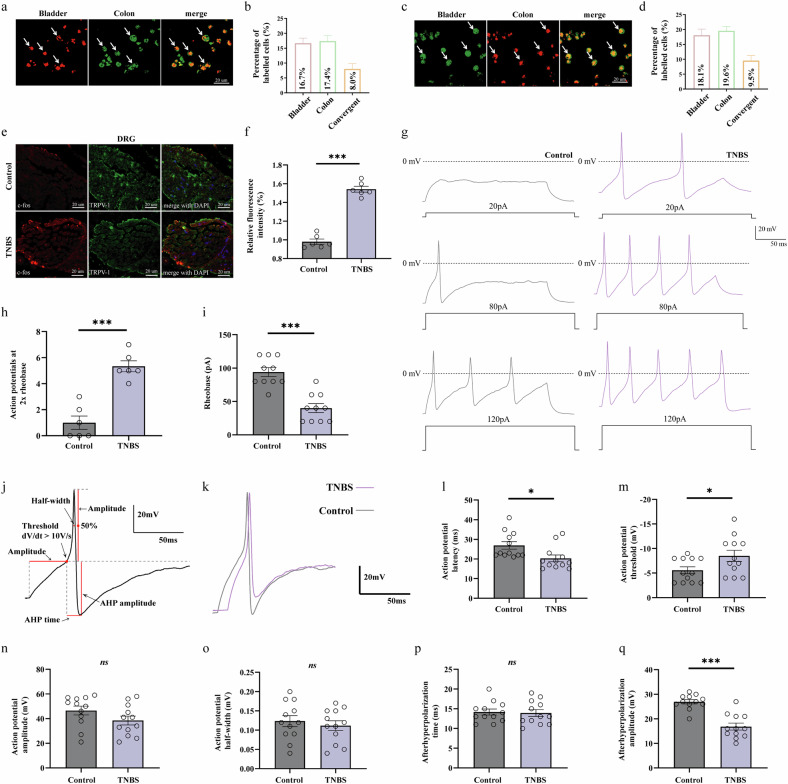
Colonic inflammation enhances the excitability of LS DRG neurons that co-innervate the colon and bladder. **a**–**d** Retrograde neuronal tracing identifies dichotomized neurons innervating both the colon and bladder in LS DRGs. Tracers were injected into the colon wall (FB, green) and bladder wall (DiI, red). Representative immunofluorescence images from L6 (**a**) and S1 (**c**) DRG sections, showing DiI-labeled (bladder-innervating), FB-labeled (colon-innervating) and double-labeled (dichotomized, orange) neurons. Quantification of the percentage of labeled neurons relative to the total number of DRG neurons in L6 (**b**) and S1 (**d**) ganglia. **e** Representative immunofluorescence images showing increased c-Fos (red) expression in TRPV1-positive (green, a marker for nociceptive neurons) DRG neurons from TNBS-treated mice compared with controls. DAPI (blue) indicates cell nuclei. Scale bar, 20 μm. **f** Relative fluorescence intensity of c-Fos in DRG sections. **g** Whole-cell current-clamp recordings from mice DRG neurons, showing AP firing in response to increasing current injections (20–120 pA). **h** DRG neurons in the TNBS-induced group displayed an increased number of APs at 2× rheobase. **i** DRG neurons in the TNBS-induced group displayed increased neuronal excitability, indicated by reduced rheobase compared with neurons in the normal group. **j** A representative AP illustrating the measurement of AP parameters. **k** Representative single APs of DRG neurons evoked by rheobase currents from control and TNBS-induced groups. **l**–**q** Summary data showing the AP latency (**l**), AP threshold (**m**), AP amplitude (**n**), AP half-width (**o**), AHP time (**p**) and AHP amplitude (**q**) from the single APs evoked by rheobase currents. Data are presented as mean ± s.e.m. The statistical difference of various indicators between the two groups was evaluated using a two-tailed unpaired Student’s *t*-test, and a statistically significant difference was defined when *P* < 0.05. Statistical significance: *P < 0.05; **P < 0.01; ***P < 0.001.

To first establish the anatomical basis for gut–bladder viscero-visceral crosstalk, we performed dual retrograde neuronal tracing by injecting FB into the colon wall and DiI into the bladder wall. In the LS DRGs, we identified a notable population of dichotomized neurons that were double-labeled with both tracers (Fig. 2a, c). Quantification showed these co-innervating neurons were present in both the L6 and S1 ganglia (Fig. 2b, d), confirming a direct anatomical circuit linking the two organs. This anatomical finding is consistent with previous tracing studies^38^. Given this shared pathway, we hypothesized that these neurons are key mediators of the comorbidity in our TNBS model. We first investigated their activation state using immunofluorescence on LS DRG sections. In TNBS-treated mice, we observed a marked increase in the expression of c-Fos, a marker of recent neuronal activity (Fig. 2e, f). Notably, many of these activated, c-Fos-positive neurons also expressed TRPV1, a marker for nociceptive sensory neurons (Fig. 2e), suggesting that the nociceptive population was preferentially activated.

To determine if this molecular activation signature translated to functional changes, we performed whole-cell patch-clamp recordings on acutely dissociated LS DRG neurons. We found that neurons from TNBS-treated mice were indeed hyperexcitable. They fired significantly more APs in response to a series of incremental depolarizing current injections compared with neurons from control mice (Fig. 2g). This hyperexcitable phenotype was further confirmed by two key metrics: a greater number of APs fired in response to a 2× rheobase current stimulus in the TNBS group (Fig. 2h) and a significantly lower rheobase (the minimum current required to elicit an AP) (Fig. 2i). We next analyzed the properties of single APs evoked by a rheobase current (Fig. 2j, k). Neurons from the TNBS group exhibitedmarked alterations indicative of increased excitability. Specifically, compared with control neurons, they showed a shorter AP latency (Fig. 2l), a lower AP threshold (Fig. 2m) and a substantially altered afterhyperpolarization (AHP) amplitude (Fig. 2q). By contrast, the AP amplitude (Fig. 2n), half-width (Fig. 2o) and AHP duration (Fig. 2p) were not significantly different between the two groups.

Collectively, these anatomical, molecular and electrophysiological data demonstrate that TNBS-induced colitis triggers a state of pronounced hyperexcitability in the LS DRG neurons, particularly within the nociceptive population that co-innervates the gut and bladder, providing a cellular basis for the observed VH and bladder dysfunction.

### Gut microbiota dysbiosis characterized by *A. muciniphila* enrichment is a shared feature of patients with OAB–IBS and the TNBS mouse model

**Fig. 3:**
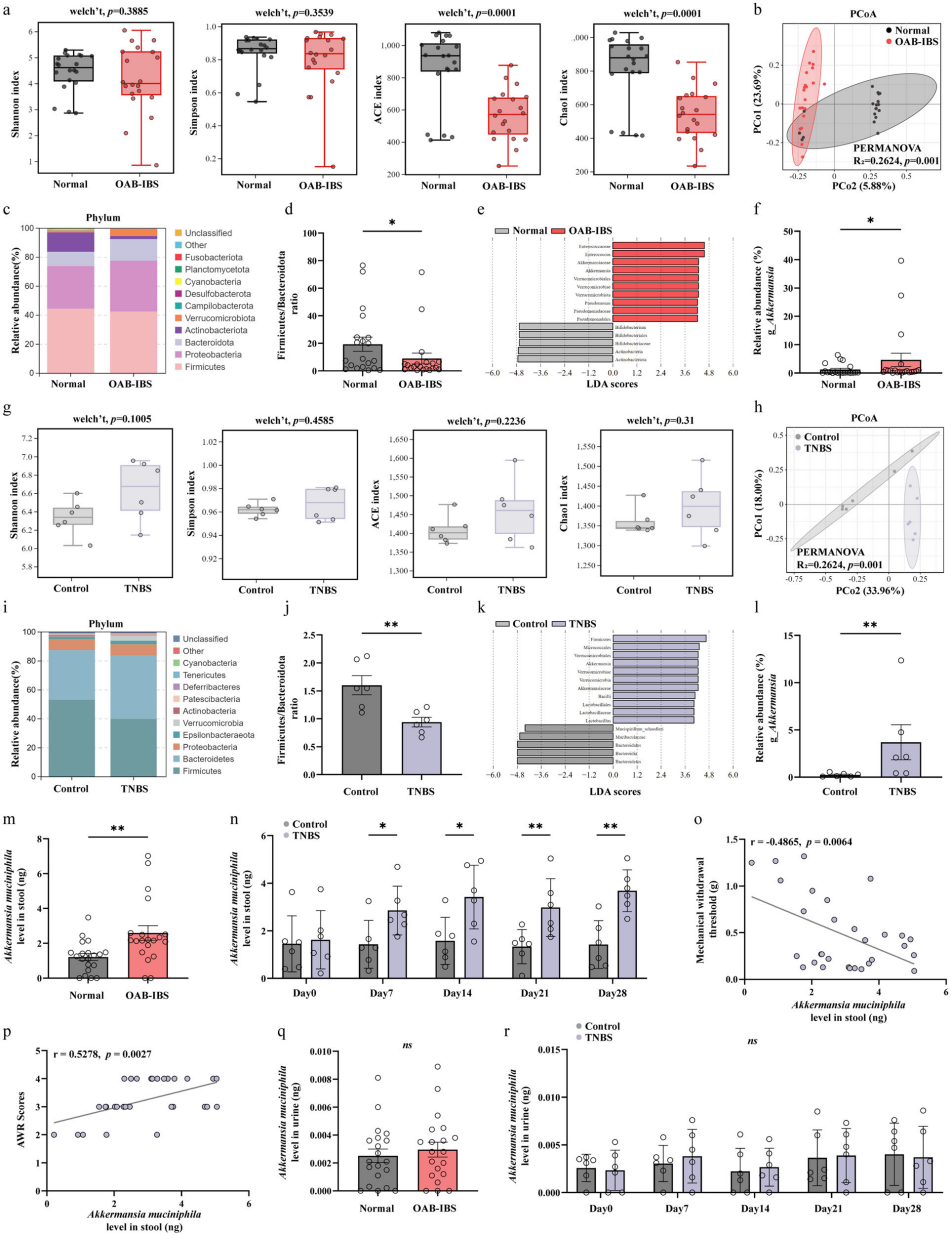
OAB–IBS is associated with gut microbiota dysbiosis characterized by the enrichment of *A. muciniphila* in both humans and a TNBS-induced mice model. **a** Comparison of α-diversity between normal and OAB–IBS groups, as measured by Shannon, Simpson, ACE and Chao1 indices. **b** PCoA plot based on Bray–Curtis dissimilarity. **c** Stacked bar plot showing the relative abundance of dominant bacterial phylum. **d** Comparison of the Firmicutes-to-Bacteroidota ratio. **e** LEfSe analysis. **f** Box plot showing the significantly higher relative abundance of the genus *Akkermansia* in patients with OAB–IBS. **g** Comparison of α-diversity between control and TNBS mice. **h** PCoA plot showing distinct microbial community structures. **i** Relative abundance of dominant bacterial phylum. **j** Comparison of the Firmicutes-to-Bacteroidota ratio. **k** LEfSe analysis. **l**, Box plot confirming the significantly higher relative abundance of the genus *Akkermansia* in TNBS mice. **m**–**p** Fecal *A. muciniphila* enrichment correlates with disease phenotype. **m** Quantification of *A. muciniphila* DNA levels by qPCR in fecal samples from normal controls and patients with OAB–IBS. **n** Time-course analysis of fecal *A. muciniphila* DNA levels. **o** Spearman correlation analysis revealing a significant negative correlation between fecal *A. muciniphila* levels and the somatic mechanical threshold (von Frey test). **p** Spearman correlation analysis showing a significant positive correlation between fecal *A. muciniphila* levels and VH (AWR scores to CRD). **q**, **r** Assessment of *A. muciniphila* in urine to exclude urinary tract colonization. Quantification of *A. muciniphila* DNA levels by qPCR in urine samples from normal controls and patients with OAB–IBS, showing no significant difference or presence (**q**). Time-course analysis of urine *A. muciniphila* DNA levels, showing no detectable levels at any time point (**r**). Data are presented as box plots or mean ± s.e.m. as appropriate. *n* = 20 for human subjects and *n* = 6 for mice per group. The statistical difference of various indicators between the two groups was evaluated using a two-tailed unpaired Student’s *t*-test, and a statistically significant difference was defined when *P* < 0.05. Statistical significance: *P < 0.05; **P < 0.01; ***P < 0.001.

Given that alterations in the gut microbiota are frequently associated with VH^39,40^, we first characterized the fecal microbial composition of patients with OAB–IBS (*n* = 20) and age-matched normal controls (*n* = 20) using 16S rDNA sequencing. While α-diversity as measured by the Shannon and Simpson indices did not differ, the ACE and Chao1 indices, which reflect community richness, were significantly different between the groups (Fig. 3a). β-Diversity analysis based on Bray–Curtis dissimilarity revealed a distinct and significant separation in the overall microbial community structure (permutational multivariate analysis of variance, *P* = 0.001; Fig. 3b). At the phylum level, the relative abundance of Verrucomicrobia was notably increased in the OAB–IBS group (Fig. 3c), and we also observed a significantly lower Firmicutes-to-Bacteroidota ratio in these patients (Fig. 3d). To identify key differential taxa, we performed linear discriminant analysis effect size (LEfSe) analysis, which highlighted the genus *Akkermansia* as the prominent biomarker enriched in the OAB–IBS cohort (Fig. 3e). Quantitative comparison confirmed this finding, showing a significantly higher relative abundance of *Akkermansia* in patients with OAB–IBS (Fig. 3f).

To determine if our TNBS-induced model of gut–bladder comorbidity recapitulated these clinical findings, we performed a parallel analysis of the murine gut microbiota. Remarkably, we observed very similar alterations. There was no change in α-diversity between control and TNBS-treated mice (Fig. 3g), but a clear separation in β-diversity was evident (permutational multivariate analysis of variance, *P* = 0.001; Fig. 3h). Consistent with the human data, Verrucomicrobia was the dominant phylum and the genus *Akkermansia* was significantly enriched in the TNBS group, as shown by relative abundance plots and LEfSe analysis (Fig. 3i, k, l). Similarly, the Firmicutes-to-Bacteroidota ratio was significantly different in TNBS-treated mice (Fig. 3j).

Given that the genus *Akkermansia* is predominantly represented by the well-characterized species *A. muciniphila* and it was identified as the key biomarker in both cohorts, we next sought to specifically quantify this species and correlate its presence with disease phenotype. Using species-specific quantitative PCR (qPCR), we confirmed that the fecal DNA levels of *A. muciniphila* were significantly elevated in patients with OAB–IBS compared with healthy controls (Fig. 3m). To validate the specificity of this association, we further analyzed the correlation between key differential taxa and clinical symptoms. Spearman correlation analysis revealed that *Akkermansia* (and the family Akkermansiaceae) exhibited a robust positive correlation with all symptom scores (IBS-SSS, OABSS and VSI), whereas other enriched taxa such as Enterococcaceae showed no significant correlation (Supplementary Fig. 3a). Furthermore, qPCR analysis of congeneric species (*A. glycaniphila* and *A. biwaensis*) showed no significant differences between groups (Supplementary Fig. 3b, c), confirming that the dysbiosis is specifically driven by *A. muciniphila*.

Considering the potential involvement of neuroactive metabolites, we also quantified fecal serotonin (5-HT) levels in the clinical cohort. Patients with OAB–IBS exhibited significantly elevated fecal 5-HT concentrations compared with controls (Supplementary Fig. 4a). Importantly, these 5-HT levels correlated positively with both the abundance of *A. muciniphila* and clinical symptom scores (Supplementary Fig. 4b–e), suggesting a potential mechanistic link between this bacterium and serotonergic signaling.

In the mouse model, a time-course analysis showed a progressive and substantial increase in *A. muciniphila* levels in TNBS-treated mice over the 28-day period (Fig. 3n). Most importantly, this fecal enrichment correlated directly with hypersensitivity phenotypes. Spearman correlation analysis revealed a significant negative correlation between fecal *A. muciniphila* and the mechanical withdrawal threshold (von Frey test) (Fig. 3o) and a significant positive correlation with VH as measured by AWR scores to CRD (Fig. 3p).

To exclude the possibility that the observed bladder dysfunction was due to direct urinary tract colonization rather than a neurally mediated gut–bladder axis, we performed species-specific qPCR for *A. muciniphila* on urine samples. We found no significant difference in *A. muciniphila* DNA levels in urine from patients with OAB–IBS compared with healthy controls (Fig. 3q). Similarly, *A. muciniphila* DNA was undetectable in the urine of TNBS-treated mice at all time points (Fig. 3r).

Taken together, these parallel findings in both humans and mice strongly implicate the fecal enrichment of *A. muciniphila* as a key microbial factor in the pathophysiology of gut–bladder comorbidity.

### Transplantation of *A. muciniphila* or microbiota derived from patients with OAB–IBS exacerbates gut–bladder axis dysfunction

**Fig. 4:**
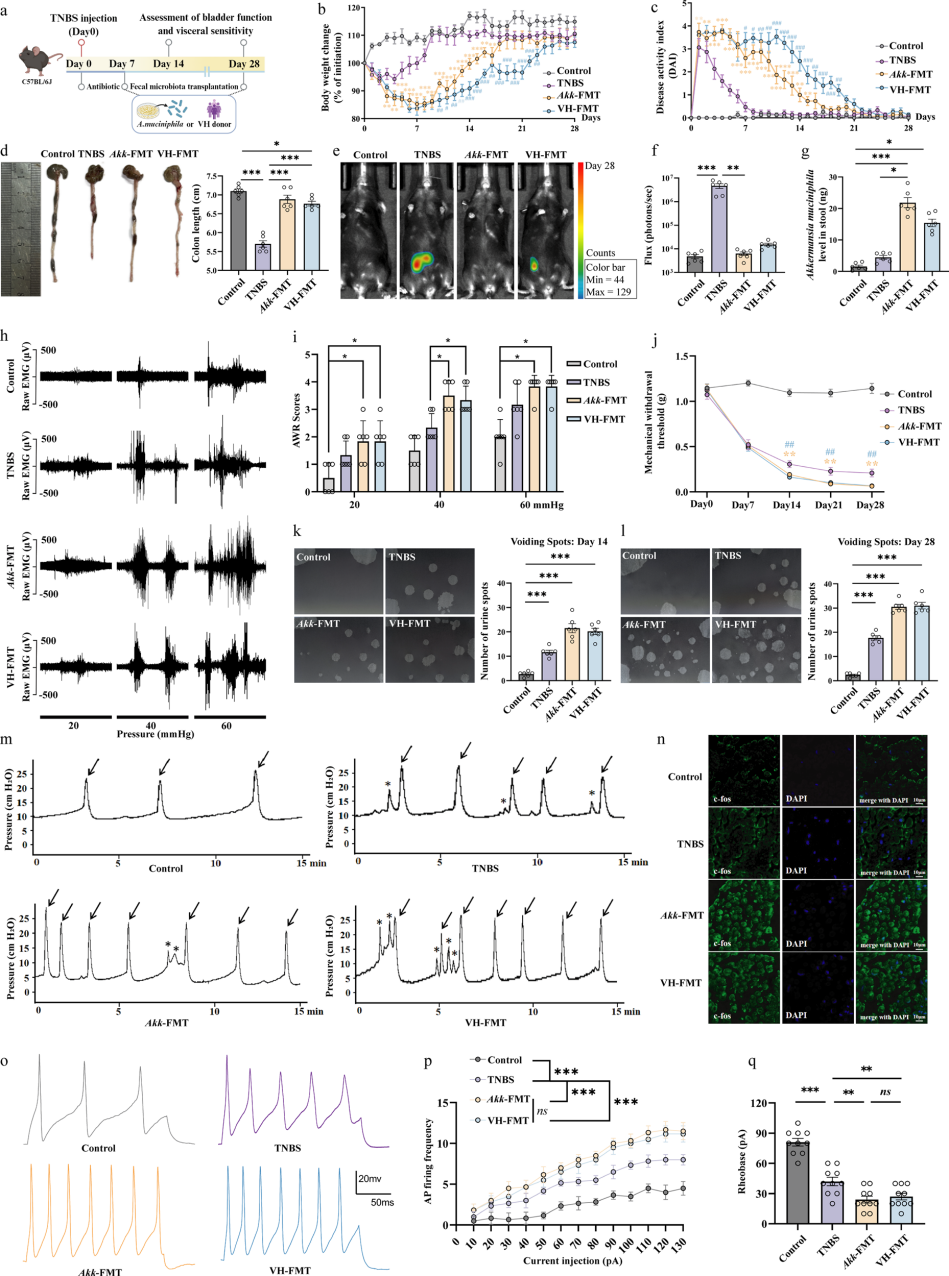
Transplantation of *A. muciniphila* or microbiota derived from patients with OAB–IBS exacerbates VH and bladder dysfunction by promoting DRG neuron hyperexcitability. **a** Schematic diagram of the experimental design. Mice were pretreated with an antibiotic cocktail (ABX) for 7 days to deplete resident gut microbiota before receiving a single gavage of *A. muciniphila* (Akk-FMT group) or fecal microbiota from patients with OAB–IBS (VH-FMT group). Control and TNBS groups received vehicle. **b**–**f** Assessment of colitis severity; ^#^*P* < 0.05; ^##^*P* < 0.01; ^###^*P* < 0.001 relative to VH-FMT group; *P < 0.05; **P < 0.01; ***P < 0.001 relative to Akk-FMT group. Comparison of body weight changes (**b**), DAI scores (**c**), colon length (**d**) and representative in vivo bioluminescence images (**e**) among the four groups. Flux values show the intestinal inflammation in four groups of mice (**f**). **g** qPCR analysis confirming the successful colonization and elevated levels of *A. muciniphila* in the Akk-FMT and VH-FMT groups compared with control and TNBS groups. **h**–**j** Evaluation of VH; ^#^*P* < 0.05; ^##^*P* < 0.01; ^###^*P* < 0.001 relative to VH-FMT group; *P < 0.05; **P < 0.01; ***P < 0.001 relative to the Akk-FMT group. Representative EMG traces of the VMR to CRD (**h**). Quantification of AWR scores (**i**). von Frey test (**j**). **k** Representative voiding-spot assay images and quantification of urine spots on day 14. **l** Corresponding images and quantification on day 28. **m** Representative continuous cystometry records on day 28. **n** Representative immunofluorescence images of c-Fos expression in LS DRG sections. **o** Representative whole-cell current-clamp recordings showing AP firing. **p**, **q** The AP firing frequency (**p**) and rheobase (**q**) of DRG neurons in current-clamp recording. The schematic diagram in panel a was created by the authors using BioRender (https://biorender.com) in 2025.

To directly test the causal role of the gut microbiota in mediating gut–bladder crosstalk^41^, we performed FMT experiments. Mice were pretreated with antibiotics to deplete resident microbiota and then divided into four groups: control, TNBS, *A. muciniphila* gavage (Akk-FMT) and FMT from patients with OAB–IBS (VH-FMT) (Fig. 4a). To rule out the possibility that the transplantation procedure itself induces adverse effects, we performed a parallel experiment using fecal microbiota from healthy donors (HC-FMT). As shown in Supplementary Fig. 5, HC-FMT treatment significantly ameliorated TNBS-induced somatic and visceral phenotypes without causing adverse effects, confirming that the exacerbated symptoms observed in the Akk-FMT and VH-FMT groups are specific to the pathogenic microbiota.

We first assessed the impact of these interventions on the resolution of colitis. Interestingly, while the Akk-FMT and VH-FMT groups exhibited a delayed recovery in both body weight and DAI scores compared with the TNBS-only group (Fig. 4b, c), their colon length on day 28 was significantly longer than that of the TNBS group and was not different from the healthy control group, suggesting an amelioration of structural damage (Fig. 4d). Meanwhile, in vivo bioluminescence imaging showed that local colonic inflammation levels were comparable among the three disease groups (Fig. 4e, f). Furthermore, histological and molecular analysis of the colon tissue on day 28 revealed that while the TNBS group still exhibited residual inflammation, both the Akk-FMT and VH-FMT groups showed a marked resolution of colitis, with histology and inflammatory marker levels comparable to the control group (Supplementary Fig. 6c, d). qPCR analysis confirmed the successful and significant enrichment of *A. muciniphila* in the fecal samples of both the Akk-FMT and VH-FMT groups (Fig. 4g). We next assessed whether these microbiota interventions impacted VH. Indeed, both the Akk-FMT and VH-FMT groups exhibited a significantly exacerbated pain-related phenotype compared with the TNBS-only group. This was demonstrated by more pronounced VMR to CRD, higher AWR scores and a lower mechanical withdrawal threshold in the von Frey test, particularly at the day 14 and 28 time points (Fig. 4h–j).

Similarly, we investigated the effects of microbiota transplantation on bladder function. Importantly, pathological analysis of the bladders on day 28 revealed no signs of cystitis, such as inflammatory infiltration or altered histology, in any of the experimental groups (Supplementary Fig. 6a, b). The OAB phenotype was also significantly worsened in the transplantation groups. Both the Akk-FMT and VH-FMT groups displayed a higher number of urine spots in the voiding-spot assay on days 14 and 28 compared with the TNBS group (Fig. 4k, l). Furthermore, continuous cystometry on day 28 revealed a markedly increased frequency of bladder contractions in both the Akk-FMT and VH-FMT groups (Fig. 4m), which was confirmed by quantitative analysis showing a significant increase in VCs (Supplementary Fig. 6e).

Finally, to determine the underlying neural mechanism for this exacerbated phenotype, we examined the activation state and excitability of LS DRG neurons. Immunofluorescence analysis revealed that c-Fos expression was significantly higher in the DRG sections of both the Akk-FMT and VH-FMT groups compared with the TNBS group (Fig. 4n and Supplementary Fig. 6f). This was functionally corroborated by whole-cell patch-clamp recordings (Fig. 4o). Neurons from both the Akk-FMT and VH-FMT groups exhibited a significantly lower rheobase and a higher AP firing frequency compared with neurons from the TNBS group, indicating a state of profound hyperexcitability (Fig. 4p, q). Collectively, these results provide direct causal evidence that *A. muciniphila* and the broader OAB–IBS dysbiotic microbiota can actively exacerbate VH and bladder dysfunction by amplifying the hyperexcitability of shared sensory neurons. This effect occurs without inducing local bladder pathology and, paradoxically, alongside an improved resolution of gut inflammation, strongly pointing toward a noninflammatory, signaling-based mechanism.

### *A. muciniphila* supernatant directly activates DRG neurons and is enriched in tryptophan metabolites

**Fig. 5:**
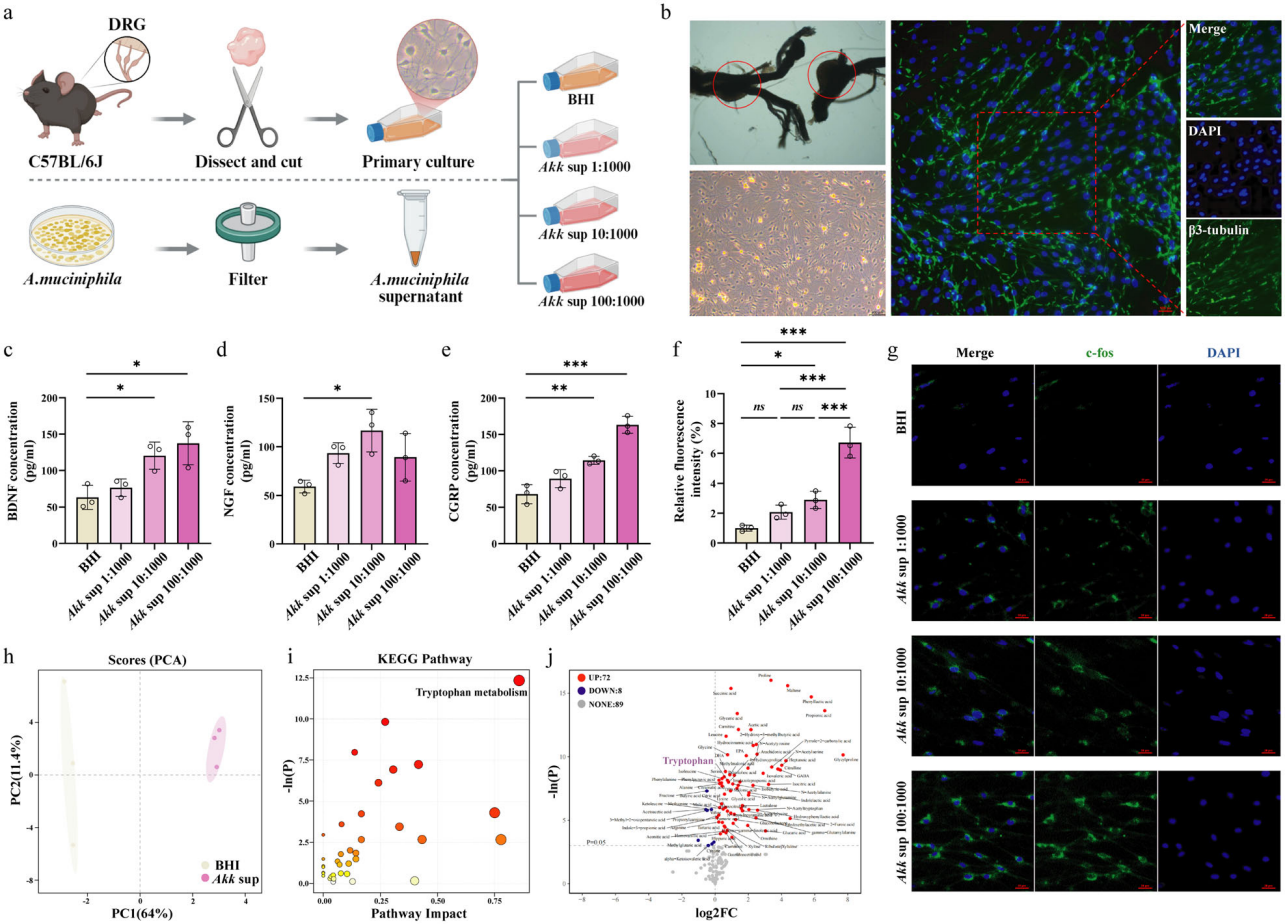
*A. muciniphila* supernatant, enriched in tryptophan metabolites, directly promotes an activated and sensitized state in primary DRG neurons. **a** Schematic diagram of the in vitro experimental workflow, illustrating the isolation of primary DRG neurons and their subsequent treatment with *A. muciniphila* supernatant (Akk sup). **b** Representative image of cultured primary DRG neurons, identified by the neuronal marker β-III tubulin (green). **c**–**e** ELISA quantification of neurotrophic and neuropeptide factors in the culture medium. *A. muciniphila* supernatant dose-dependently increased the levels of BDNF (**c**) and NGF (**d**) and also elevated CGRP levels (**e**). **f**, **g** Assessment of neuronal activation by c-Fos immunofluorescence. Quantification shows a dose-dependent increase in the percentage of c-Fos-positive neurons following treatment (**f**). Representative images of c-Fos staining (green) in DRG neurons (**g**). **h**–**j** Targeted metabolomic analysis of the *A. muciniphila* supernatant. PCA plot showing a clear metabolic distinction between *A. muciniphila* supernatant and BHI medium (**h**). KEGG pathway analysis indicating significant enrichment of the tryptophan metabolism pathway (**i**). Volcano plot highlighting differentially abundant metabolites, with tryptophan being significantly elevated in the *A. muciniphila* supernatant compared with the BHI control (**j**). Data are presented as mean ± s.e.m. The statistical difference of various indicators between the two groups was evaluated using a two-tailed unpaired Student’s *t*-test, and a statistically significant difference was defined when *P* < 0.05. For multigroup comparisons involving different doses, statistical significance was determined using one-way ANOVA followed by Tukey’s multiple comparisons test. Statistical significance: *P < 0.05; **P < 0.01; ***P < 0.001. The schematic diagram in panel a was created by the authors using BioRender (https://biorender.com) in 2025.

To investigate if *A. muciniphila* could directly influence sensory neuron activity, we established an in vitro coculture system where primary DRG neurons were exposed to cell-free supernatant from *A. muciniphila* culture (Akk sup) (Fig. 5a, b). Treatment with Akk sup induced a state of neuronal activation and sensitization. Specifically, it dose-dependently increased the secretion of the neurotrophic factors BDNF and NGF, and the neuropeptide CGRP, as measured by ELISA (Fig. 5c–e). Furthermore, immunofluorescence analysis revealed a significant, dose-dependent increase in the percentage of c-Fos-positive neurons, confirming direct neuronal activation by the bacterial supernatant (Fig. 5f, g). To identify the potential neuroactive molecules within the supernatant, we performed targeted metabolomics. Principal component analysis (PCA) showed a clear distinction between the metabolic profiles of Akk sup and the BHI control medium (Fig. 5h). Crucially, Kyoto Encyclopedia of Genes and Genomes (KEGG) pathway enrichment analysis of the differential metabolites identified ‘Tryptophan metabolism’ as the most significantly altered pathway (Fig. 5i). A volcano plot highlighted tryptophan itself as one of the most significantly elevated metabolites in the Akk sup (Fig. 5j). This unbiased metabolomic profiling identified tryptophan accumulation as a key metabolic signature of *A. muciniphila*, providing a strong rationale for investigating tryptophan and its downstream serotonergic metabolites as the primary mediators of the observed neuronal activation^18^.

### *A. muciniphila*-enriched microbiota shunts gut tryptophan metabolism toward the serotonin pathway and upregulates the 5-HT_3a_ receptor in vivo

**Fig. 6:**
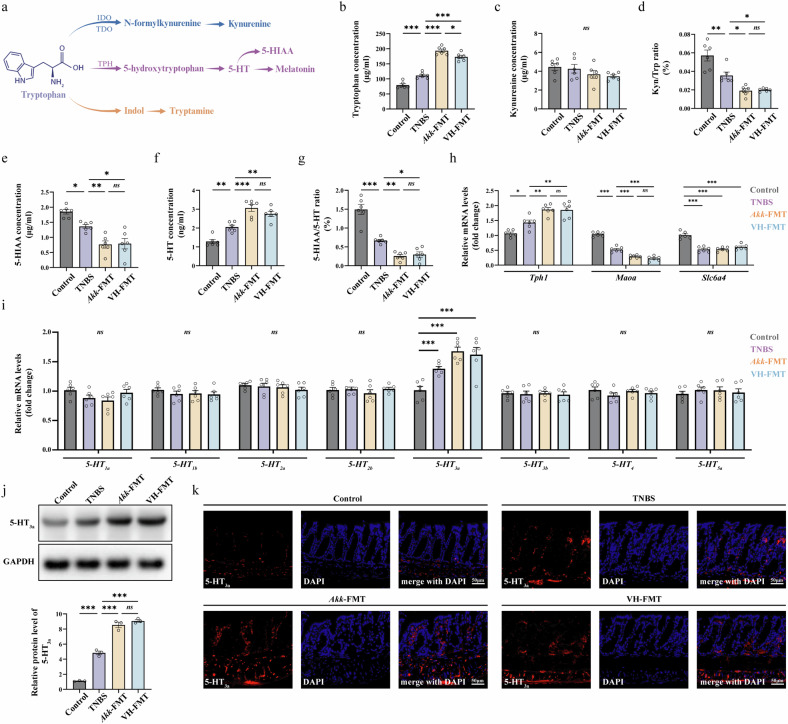
*A. muciniphila* and OAB–IBS microbiota shift gut tryptophan metabolism toward the serotonin pathway and upregulate the 5-HT_3a_ receptor. **a** Tryptophan metabolism pathways. **b**–**d** ELISA analysis of the kynurenine pathway in fecal samples. Tryptophan (Trp) (**b**) and Kynurenine (Kyn) (**c**) concentrations and the kynurenine-to-tryptophan (Trp/Kyn) ratio (**d**). **e**–**g** ELISA analysis of the serotonin pathway in fecal samples. 5-HIAA (**e**) and 5-HT (**f**) concentrations and the 5-HIAA-to-5-HT (5-HIAA/5-HT) ratio, indicating serotonin turnover (**g**). The Akk-FMT and VH-FMT groups showed significantly increased 5-HT levels and a decreased turnover ratio. **h**, qPCR analysis of genes related to 5-HT metabolism in colon tissue. Expression levels of tryptophan hydroxylase 1 (*Tph1*), the serotonin transporter (*Slc6a4*) and monoamine oxidase A (*Maoa*). **i** qPCR screening of various 5-HT receptor subtypes in colon tissue, revealing that only the 5-HT_3a_ receptor (*Htr3a*) was significantly upregulated. (**j**) Western blot analysis and quantification of 5-HT_3a_ receptor protein expression in colon tissue. Expression was the highest in the Akk-FMT and VH-FMT groups. (**k**) Representative immunofluorescence images showing increased expression and localization of the 5-HT_3a_ receptor (red) in colon sections from the four groups. DAPI (blue) stains cell nuclei. Scale bar, 50 μm. Data are presented as mean ± s.e.m. Statistical differences among groups were evaluated using one-way ANOVA followed by Tukey’s multiple comparisons test, and statistical significance was defined when *P* < 0.05. Statistical significance: *P < 0.05; **P < 0.01; ***P < 0.001. The metabolism pathway diagram in panel a was created by the authors using BioRender (https://biorender.com) in 2025.

On the basis of our in vitro findings, we next investigated how tryptophan metabolism was altered in vivo in our FMT mouse models (pathway shown in Fig. 6a). Analysis of fecal metabolites revealed that while tryptophan levels were elevated in the Akk-FMT and VH-FMT groups, there were no significant changes in kynurenine levels, resulting in an altered tryptophan-to-kynurenine ratio (Fig. 6b–d). By contrast, analysis of the serotonin pathway showed a significant increase in 5-HT levels and a correspondingly decreased 5-HIAA/5-HT turnover ratio in both the Akk-FMT and VH-FMT groups (Fig. 6e–g). To rigorously validate these ELISA-based measurements and exclude potential cross-reactivity, we performed targeted liquid chromatography–tandem mass spectrometry (LC–MS/MS) analysis on colonic tissues. As shown in Supplementary Fig. 7, mass spectrometry quantification confirmed the metabolic trends observed with ELISA, specifically demonstrating a robust and significant increase in 5-HT and tryptophan levels in both the Akk-FMT and VH-FMT groups compared with TNBS controls. This indicates a clear metabolic shift toward 5-HT production and accumulation in the gut.

To elucidate the molecular basis for this metabolic shunting, we analyzed the expression of key genes in colon tissue. Consistent with the metabolite data, the expression of tryptophan hydroxylase 1 (*Tph1*), the rate-limiting enzyme for peripheral 5-HT synthesis, was significantly upregulated in the transplantation groups. Conversely, the serotonin transporter (*Slc6a4*) and the degradation enzyme monoamine oxidase A (*Maoa*) were both downregulated (Fig. 6h). This suggests a dual-drive mechanism where *A. muciniphila* increases substrate availability (tryptophan) while simultaneously driving host transcriptional reprogramming (*Tph1* upregulation).

With elevated 5-HT levels, we next screened for its potential receptors and found that only the 5-HT_3a_ receptor gene was significantly upregulated in the colons of TNBS and FMT-treated mice (Fig. 6i). This was confirmed at the protein level by western blot and immunofluorescence, which showed a marked increase in 5-HT_3a_ receptor expression, with the highest levels observed in the Akk-FMT and VH-FMT groups (Fig. 6j, k). Crucially, to determine the cellular localization of these upregulated receptors, we performed double immunofluorescence staining and retrograde tracing. As shown in Supplementary Fig. 8, we found that the increased 5-HT_3a_ receptors were specifically localized to PGP9.5-positive nerve fibers and, more importantly, to the FB-labeled DRG neurons projecting to the colon. This confirms that the serotonergic signal targets the extrinsic sensory afferent pathway rather than acting on local epithelial cells.

Collectively, these in vitro and in vivo data delineate a mechanistic pathway wherein an *A. muciniphila*-enriched microbiota alters host tryptophan metabolism to boost gut serotonin synthesis, which in turn acts on upregulated colonic 5-HT_3a_ receptors on sensory nerve terminals to drive the neuronal hyperexcitability underlying gut–bladder dysfunction.

### Blockade of the gut-specific 5-HT_3a_ receptor or mesenteric nerve signaling reverses gut–bladder dysfunction

**Fig. 7:**
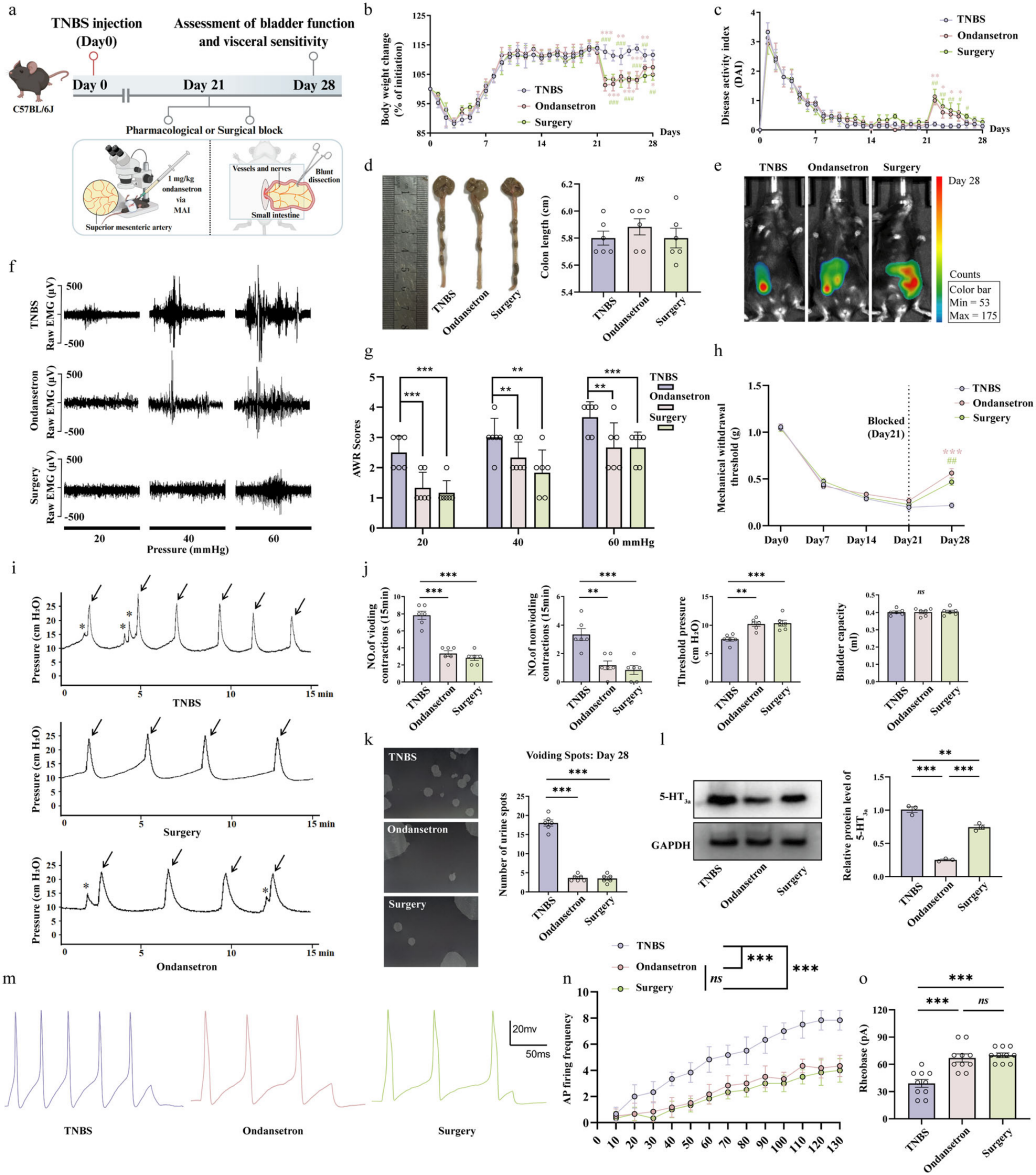
Pharmacological blockade of gut-specific 5-HT_3a_ receptors or surgical mesenteric denervation ameliorates TNBS-induced VH and bladder dysfunction. **a** Schematic diagram of the intervention strategies. On day 21 after TNBS induction, mice received either a targeted injection of the 5-HT_3a_ antagonist ondansetron into the mesenteric artery (ondansetron group) or underwent surgical denervation of the colon’s mesenteric nerves (surgery group). The TNBS group received a vehicle injection and served as the disease control. **b**–**e** Assessment of colitis severity following interventions; ^#^*P* < 0.05; ^##^*P* < 0.01; ^###^*P* < 0.001 relative to the surgery group; *P < 0.05; **P < 0.01; ***P < 0.001 relative to the ondansetron group. Body weight changes (**b**), DAI scores (**c**), colon length (**d**) and representative in vivo bioluminescence images (**e**), showing no significant differences among the three groups on the 28th day. **f** Representative EMG traces of the VMR. **g** Quantification of AWR scores during CRD. **h** von Frey test. **i**–**k** Both interventions reversed the OAB phenotype. Representative continuous cystometry records on day 28 (**i**). The urodynamic parameters evaluated included VCs (arrows), non-VCs (stars), TP and bladder capacity (**j**). Representative voiding-spot assay images and quantification of urine-spot numbers on day 28 (**k**). **l** Western blot analysis of 5-HT_3a_ receptor expression in the colon. **m**–**o**, Both interventions normalized DRG neuron excitability. Representative whole-cell current-clamp recordings showing AP firing (**m**). The AP firing frequency (**n**) and rheobase (**o**) of DRG neurons in current-clamp recording. The schematic diagram in panel a was created by the authors using BioRender (https://biorender.com) in 2025.

To definitively establish that the observed gut–bladder comorbidity is driven by pathological signaling transmitted via the gut–DRG neural axis, we used two intervention strategies: pharmacological blockade of the gut-specific 5-HT_3a_ receptor with ondansetron and surgical denervation of the colon’s mesenteric nerves (Fig. 7a). To ensure that any functional improvements were not due to an amelioration of the underlying colitis, we first assessed disease markers. On day 28, we found no significant differences in body weight, DAI scores, colon length or colonic inflammatory signals among the TNBS, ondansetron and surgery groups (Fig. 7b–e). This indicates that the interventions did not alter the colonic inflammatory state itself. Having established this, we next tested the effects of these interventions on the functional consequences. Both pharmacological blockade with ondansetron and surgical denervation significantly alleviated visceral and somatic hypersensitivity. This was evidenced by reduced VMRs, lower AWR scores during CRD and a higher mechanical withdrawal threshold, compared with the TNBS vehicle control group (Fig. 7f–h). Similarly, both interventions successfully reversed the OAB phenotype. Representative continuous cystometry records showed a marked reduction in bladder contraction frequency, and the voiding-spot assay confirmed a normalized urination pattern in both the ondansetron and surgery groups (Fig. 7i–k). Crucially, the efficacy of mesenteric denervation—which physically interrupts the neural connection while leaving the systemic circulation intact—provides definitive proof that the cross-organ dysfunction is mediated by a local neural circuit rather than by circulating humoral factors.

Finally, to confirm that these behavioral and functional improvements were rooted in the normalization of neuronal activity, we assessed the excitability of LS DRG neurons. Whole-cell patch-clamp recordings revealed that neurons from both ondansetron-treated and surgically denervated mice were significantly less excitable than those from the TNBS group. This was demonstrated by a higher rheobase and a lower AP firing frequency, restoring them to levels comparable to healthy controls (Fig. 7m–o). To provide further mechanistic insight, we measured 5-HT_3a_ receptor expression in the colon. Interestingly, while pharmacological blockade was associated with downstream normalization, surgical denervation did not reduce the TNBS-induced upregulation of the 5-HT_3a_ receptor (Fig. 7l). This suggests that while receptor upregulation is a local gut phenomenon, its ability to drive downstream pathology is entirely dependent on an intact afferent neural connection. Taken together, these intervention experiments provide definitive causal evidence that the gut–bladder comorbidity in this model is driven by a pathological signaling axis originating from the gut, acting through the 5-HT_3a_ receptor, and transmitted via mesenteric afferent nerves to induce a state of sensory neuron hyperexcitability.

### Blockade of the 5-HT_3a_ receptor reverses the specific pathological effects driven by *A. muciniphila*

**Fig. 8:**
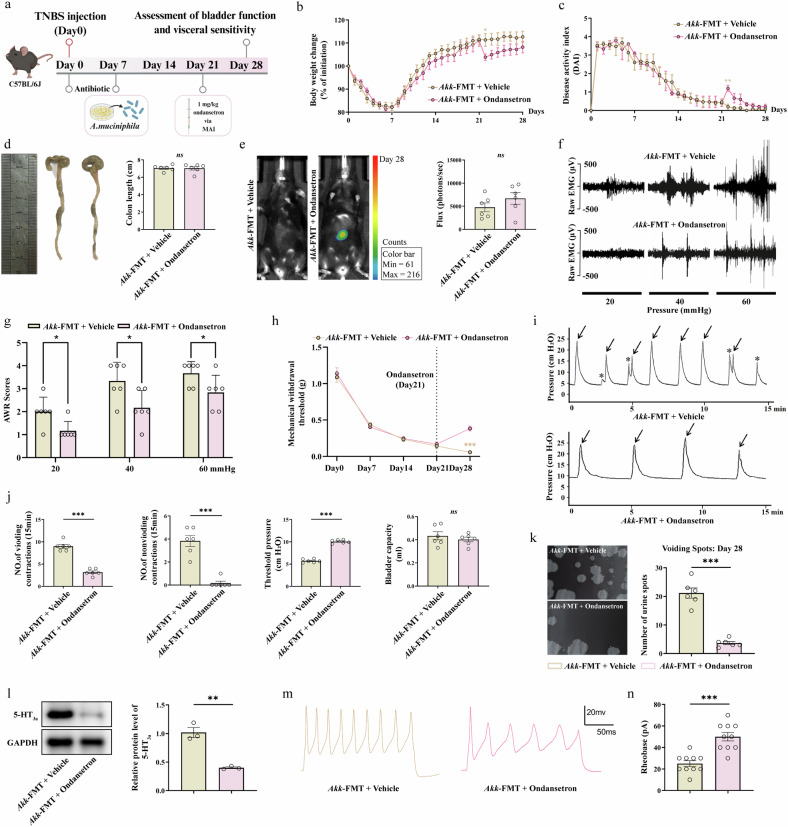
Pharmacological blockade of the 5-HT_3a_ receptor specifically abrogates the exacerbation of gut–bladder dysfunction driven by *A. muciniphila.* To confirm that the pronociceptive effects of *A. muciniphila* are mediated by the 5-HT_3a_ receptor, TNBS-treated mice gavaged with *A. muciniphila* (Akk-FMT group) were administered either vehicle or the 5-HT_3a_ antagonist ondansetron from day 21 to day 28. **a** Schematic diagram of the intervention strategies. **b**–**e** Body weight changes (**b**), DAI scores (**c**), colon length (**d**) and representative in vivo bioluminescence images (**e**). **f** Representative EMG traces of the VMR. **g** Quantification of AWR scores during CRD. **h** Abdominal withdrawal frequency in the von Frey test. **i** Representative continuous cystometry records on day 28. **j** The urodynamic parameters evaluated included VCs (arrows), non-VCs (stars), TP and bladder capacity. **k** Representative voiding-spot assay images and quantification of urine-spot numbers on day 28. **l** Western blot analysis of 5-HT_3a_ receptor expression in the colon. **m**, **n** Both interventions normalized DRG neuron excitability. **m** Representative whole-cell current-clamp recordings showing AP firing. **n** The rheobase of DRG neurons in current-clamp recording. Data are presented as mean ± s.e.m. Statistical differences among groups were evaluated using one-way ANOVA followed by Tukey’s multiple comparisons test, and statistical significance was defined when *P* < 0.05. Statistical significance: *P < 0.05; **P < 0.01; ***P < 0.001. The schematic diagram in panel a was created by the authors using BioRender (https://biorender.com) in 2025.

Finally, to provide definitive causal evidence linking our identified microbial driver (*A. muciniphila*) to the specific molecular target (5-HT_3a_R), we performed a targeted pharmacological intervention. TNBS mice from the Akk-FMT group, which exhibited the most severe phenotype, were treated with either ondansetron or vehicle (Fig. 8a). As a critical control, we first assessed the severity of colitis. The body weight and DAI scores between the ondansetron and vehicle-treated groups were largely comparable, with the exception of a transient difference on day 22, which was probably attributable to the injection procedure itself rather than a pharmacological effect on the colitis (Fig. 8b, c). Overall, there were no significant differences in colon length or inflammatory bioluminescence between the groups on day 28 (Fig. 8d, e).

Despite having no effect on the colonic inflammation, ondansetron treatment potently reversed the functional consequences of *A. muciniphila* colonization. The exacerbated visceral and somatic hypersensitivity was significantly ameliorated, as shown by representative EMG traces, lower AWR scores and a higher abdominal withdrawal frequency threshold in the von Frey test (Fig. 8f–h). Similarly, the OAB phenotype was completely abrogated. Representative continuous cystometry records showed a normalized bladder contraction frequency (Fig. 8i, j), and the voiding-spot assay confirmed a restored, healthy urination pattern in the ondansetron-treated group (Fig. 8k).

At the cellular level, this functional rescue was underpinned by the normalization of sensory neuron activity. Western blot analysis revealed that ondansetron treatment significantly reduced the *A. muciniphila*-induced upregulation of 5-HT_3a_ receptor expression in the colon (Fig. 8l). Critically, whole-cell patch-clamp recordings of LS DRG neurons demonstrated that the profound hyperexcitability in the Akk-FMT group was completely reversed by ondansetron, as evidenced by a restored rheobase and a normalized AP firing frequency (Fig. 8m, n). These findings provide the ultimate proof that *A. muciniphila* actively drives the gut–bladder axis dysfunction through the pathological activation of the 5-HT_3a_ receptor signaling pathway, solidifying this axis as a key therapeutic target.

## Discussion

In this study, we delineated a novel, detailed mechanism connecting a specific gut microorganism, *A. muciniphila*, with bladder dysfunction, providing a compelling explanation for the clinical comorbidity of IBS and OAB. We demonstrate that an *A. muciniphila*-enriched microbiota drives gut–bladder crosstalk by regulating a dual-drive modulation of host tryptophan metabolism. This metabolic shift promotes the synthesis of gut 5-HT, which in turn acts on specifically upregulated 5-HT_3a_ receptors on extrinsic sensory nerve terminals to induce a state of hyperexcitability in shared LS sensory neurons, ultimately causing a sustained OAB phenotype.

Our study began by establishing a robust ‘postinflammatory’ murine model that successfully recapitulates key features of human OAB–IBS comorbidity, a link we first confirmed through strong clinical correlations between OABSS, IBS-SSS and VSI scores in patients (Supplementary Fig. 1). A single TNBS insult induced acute colitis that resolved systemically yet left behind long-lasting VH and a clear OAB phenotype (Fig. 1). Crucially, this bladder dysfunction occurred in the complete absence of local bladder inflammation or pathology (Supplementary Figs. 2 and 6). Furthermore, we explicitly excluded urinary tract colonization by *A. muciniphila* as a confounding factor, as qPCR analysis of urine from both our patient cohort and mouse model showed no detectable levels of the bacterium (Fig. 3q, r). This finding is critical, as it models the functional nature of these disorders and strongly suggests that the bladder symptoms are not due to direct cystitis but are a neurally mediated consequence of a distant gut pathology, a hallmark of viscero-visceral crosstalk^7,9,14^.

A key and perhaps surprising finding of our study is the identification of *A. muciniphila* as a pivotal microbial player in this process. Through parallel 16S rDNA sequencing, we identified the enrichment of *Akkermansia* as the most consistent biomarker in both human patients with OAB–IBS and our TNBS mouse model (Fig. 3). The specificity of this association was further validated by the observation that only *A. muciniphila*, and not other dysbiotic taxa such as *Enterococcus*, correlated with symptom severity (Supplementary Fig. 3). This finding, while contrasting with some literature reporting *Akkermansia* depletion in IBS, highlights the context-dependent nature of this microorganism^42,43^. Our data resonate with recent high-impact studies that have challenged the universal probiotic view of *A. muciniphila*, identifying its potential pathogenic role in specific contexts, such as exacerbating food allergy in fiber-deprived mice or posttranslationally modifying IgA1 in autoimmune glomerulonephritis^44,45^. More importantly, our study reveals a striking dissociation between this microorganism’s structural and signaling roles. Our FMT experiments provided causal evidence, as transplantation of *A. muciniphila* or microbiota from patients with OAB–IBS exacerbated both VH and the OAB phenotype (Fig. 4). This functional link was further strengthened by correlations between fecal *A. muciniphila* abundance and both somatic (von Frey) and, critically, visceral (CRD) hypersensitivity measures (Fig. 3o, p). Paradoxically, this functional decline occurred even as the transplanted microbiota promoted the histological resolution of colon inflammation (Supplementary Fig. 6). This striking dissociation between pathological inflammation and functional pain strongly supports our hypothesis that *A. muciniphila*’s impact is mediated by specific signaling pathways rather than by modulating the severity of gut inflammation itself.

Our study further delineates this signaling pathway with precision. While the link between serotonin and visceral pain is established, the upstream drivers initiating this cascade in the context of dysbiosis have remained elusive. Here, we identify a novel ‘substrate–enzyme synergy’ driven by *A. muciniphila*. We first demonstrated in vitro that supernatant from *A. muciniphila*, which we found to be enriched in tryptophan, can directly activate and sensitize primary DRG neurons (Fig. 5). Moving back in vivo, we showed that the *A. muciniphila*-enriched microbiota shunts host tryptophan metabolism away from the kynurenine pathway and toward the serotonin pathway. This metabolic shift was rigorously validated by targeted LC–MS/MS, which confirmed the accumulation of tryptophan and 5-HT in the colon (Supplementary Fig. 7). A critical aspect of this pathway is the clarification that *A. muciniphila* does not synthesize 5-HT directly. 5-HT is a host neurotransmitter produced by enterochromaffin (EC) cells via the enzyme Tph1^46^. Our findings align with and extend previous studies which have linked *A. muciniphila* to the modulation of host tryptophan metabolism^47^. We therefore propose a ‘microbiota–metabolite–EC cell’ axis: *A. muciniphila* provides the tryptophan substrate (Fig. 5j), which fuels the host’s serotonergic machinery. This is strongly supported by our in vivo data showing that Akk-FMT and VH-FMT lead to a significant upregulation of host colonic *Tph1* gene expression (Fig. 6h). Concurrently, these transplants decreased the expression of the reuptake transporter (*Slc6a4*) and the degradation enzyme (*Maoa*), providing a complete molecular explanation for the observed accumulation of gut serotonin (Fig. 6h). Crucially, we identified the specific molecular target for this excess serotonin. Our screening revealed a significant and specific upregulation of only the 5-HT_3a_ receptor in the colons of mice with gut–bladder dysfunction (Fig. 6i–k). We focused our analysis of 5-HT_3a_R on the colon, as this is the site of the primary afferent nerve terminals (Fig. 2a–d), where the signal is first received and transduced. Using retrograde tracing combined with immunofluorescence, we definitively localized these upregulated receptors to the extrinsic sensory afferents projecting to the DRG, rather than solely to the intrinsic enteric nervous system (Supplementary Fig. 8). The 5-HT_3a_ receptor, a ligand-gated ion channel, is perfectly suited to transduce this chemical signal (5-HT) into an electrical signal (AP) that propagates along the afferent nerve to the DRG cell body. The functional necessity of this entire peripheral signaling cascade was definitively proven by our intervention experiments (Figs. 7 and 8). Both surgical denervation (severing the neural connection) and pharmacological blockade (inhibiting the receptor) completely reversed the DRG hyperexcitability and the disease phenotypes.

A key finding of our surgical intervention requires clarification. Our finding that mesenteric denervation ameliorated hypersensitivity seems counterintuitive to the concept of nerve injury causing pain. However, our procedure must be distinguished from nerve injury-induced pain. Our procedure was one of de-afferentation—a complete severing of the signal input from the gut. The fact that unplugging the colon normalized DRG excitability (Fig. 8p, q) provides powerful proof that the hyperexcitability was not autonomous within the ganglia but was contingent upon a continuous, pathological afferent drive from the 5-HT-rich gut environment. Furthermore, the efficacy of this nerve-specific intervention effectively rules out the possibility that the cross-organ sensitization is mediated by circulating systemic factors, reinforcing the neural nature of the crosstalk.

The therapeutic implications of these findings are significant and directly supported by both our clinical correlations and definitive preclinical interventions. Ondansetron, a widely used 5-HT_3a_ receptor antagonist, could potentially be repurposed for treating OAB–IBS comorbidity^48,49^, offering a mechanism-based therapeutic strategy that our data suggest can be targeted to the gut. Furthermore, interventions aimed at modulating the gut microbiota, perhaps by selectively reducing *A. muciniphila* abundance or, more subtly, altering its metabolic output of tryptophan, could represent a novel upstream approach to restoring gut–bladder homeostasis.

Despite these insights, our study has limitations. For instance, while ondansetron was administered via the mesenteric artery to enhance gut specificity, we cannot definitively exclude the possibility of systemic or central nervous system effects. This limitation is noted; however, our findings from the surgical denervation experiment provide important context. The observation that this purely peripheral intervention, which only severs the afferent nerve connection, largely recapitulated the therapeutic effects of ondansetron lends significant support to the hypothesis that the pathological signaling is primarily mediated at the peripheral gut–nerve interface. Nonetheless, a potential contribution of central 5-HT_3a_ receptors cannot be entirely ruled out. While our FMT experiments provide strong causal evidence for the role of the dysbiotic microbiota, future studies using gnotobiotic mice colonized with *A. muciniphila* alone are needed to confirm its specific, independent role and dissect the context-dependency of its effects. In addition, clinical trials are necessary to validate the therapeutic efficacy of targeting the 5-HT_3a_ receptor in patients with OAB–IBS.

In conclusion, this study reveals a detailed mechanistic pathway by which the gut bacterium *A. muciniphila* orchestrates gut–bladder crosstalk. By regulating host tryptophan metabolism toward the serotonin pathway via a substrate–enzyme synergistic mechanism, it generates a pathological signal that, through the 5-HT_3a_ receptor on extrinsic afferents, sensitizes shared neurons, leading to bladder dysfunction. This work not only provides a new pathophysiological framework for understanding OAB–IBS comorbidity but also identifies the gut-specific 5-HT_3a_ receptor as a key, druggable node for therapeutic intervention, while also highlighting the complex, context-dependent role of *A. muciniphila* in health and disease.

## Supplementary information

Supplementary Information Supplementary Table 1. The comparisons of demographic characteristics and symptom scores between patients with OAB–IBS and asymptomatic controls. Supplementary Table 2. Primer sequences used in this study. Supplementary Fig. 1. Relationship between bladder, gut and visceral sensitivity in patients with OAB–IBS. Supplementary Fig. 2. Histological and inflammatory marker analysis of the bladder and colon in the TNBS-induced colitis model. Supplementary Fig. 3. Correlation analysis of gut microbiota with clinical symptoms and species specificity validation. Supplementary Fig. 4. Fecal serotonin levels are elevated in patients with OAB–IBS and correlate with *A. muciniphila* abundance and symptom severity. Supplementary Fig. 5. Healthy control microbiota transplantation (HC-FMT) ameliorates TNBS-induced somatic and visceral phenotypes. Supplementary Fig. 6. FMT exacerbates bladder dysfunction despite promoting colitis resolution and not inducing cystitis. Supplementary Fig. 7. Validation of colonic tryptophan pathway metabolites using targeted LC–MS/MS. Supplementary Fig. 8. Anatomical localization of 5-HT_3a_ receptors in the colon and DRG.

## Data Availability

All data used in this study are present in this Article and its Supplementary Information. Additional data and materials can also be requested from corresponding author. The raw data of 16S rDNA gene sequencing have been submitted to the Short Read Archive (PRJNA1222454).
