# Supplementary material for: Akkermansia muciniphila drives viscero-visceral crosstalk via 5-HT3aR-mediated sensitization of dichotomizing gut–bladder neurons
Source: Exp Mol Med. 2026 May 8;58(5):1608–25. doi: 10.1038/s12276-026-01720-4 (PMC13234170; doi:10.1038/s12276-026-01720-4)
Supplement: Supplementary file 1 — Supplementary Table 1. The comparisons of demographic characteristics and symptom scores between patients with OAB–IBS and asymptomatic controls. Supplementary Table 2. Primer sequences used in this study. Supplementary Fig. 1. Relationship between bladder, gut and visceral sensitivity in patients with OAB–IBS. Supplementary Fig. 2. Histological and inflammatory marker analysis of the bladder and colon in the TNBS-induced colitis model. Supplementary Fig. 3. Correlation analysis of gut microbiota with clinical symptoms and species specificity validation. Supplementary Fig. 4. Fecal serotonin levels are elevated in patients with OAB–IBS and correlate with A. muciniphila abundance and symptom severity. Supplementary Fig. 5. Healthy control microbiota transplantation (HC-FMT) ameliorates TNBS-induced somatic and visceral phenotypes. Supplementary Fig. 6. FMT exacerbates bladder dysfunction despite promoting colitis resolution and not inducing cystitis. Supplementary Fig. 7. Validation of colonic tryptophan pathway metabolites using targeted LC–MS/MS. Supplementary Fig. 8. Anatomical localization of 5-HT3a receptors in the colon and DRG. [file 12276_2026_1720_MOESM1_ESM.pdf]

Supplementary Table 1. **The comparisons of demographic characteristics and symptom scores between OAB-IBS patients and Normal controls.**

Characteristic	OAB-IBS (N=20)	Normal controls (N=20)	*p* Value[e]
Age, years	45.25 ± 11.43	41.30 ± 12.25	0.2984
BMI, kg/m^2	20.45 ± 2.965	21.13 ± 3.175	0.4856
Hypertension, N (%)	4 (20)	2 (10)	0.3758
Diabetes, N (%)	3 (15)	2 (10)	0.6326
IBS subtype, N (%)			
IBS-M (Mixed)	8 (40)	/	
IBS-D (Diarrhea)	6 (30)	/	
IBS-C (Constipation)	5 (25)	/	
IBS-U (Unsubtyped)	1 (5)	/	
OABSS, scores	8.0 (4.0,7.0)	0.85 (0.0,1.0)	<0.001
OAB-V8, scores	17.30 (8.50,24.50)	1.75 (0.25,2.75)	<0.001
IBS-SSS, scores	292.0 (200.0,375.0)	121 (100.0,140.0)	<0.001
VSI, scores	44.60 (29.50,61.25)	1.05 (0.0,2.0)	<0.001
Bristol Stool Scale, scores	4.95 (3.25,6.00)	3.05 (2.25,4.00)	0.0061

BMI, body mass index. [e]*p* Values were calculated using Wilcoxon rank-sum test for continuous and Fisher's exact test for categorical variables. The data are expressed as the means ± standard deviations or medians (interquartile ranges).

Supplementary Table 2. Primer sequences used in this study.

Genes	Forward primer (5′ - 3′)	Reverse primer (5′ - 3′)
TNF-α	AGGGTCTGGGCCATAGAACT	CCACCACGCTCTTCTGTCTAC
IL-1β	TGCCACCTTTTGACAGTGATG	AAGGTCCACGGGAAAGACAC
IL-6	GAGCCCACCAAGAACGATAG	TCCACGATTTCCCAGAGAAC
cxcl-2	TCCAGAGCTTGAGTGTGACG	CTTTGGTTCTTCCGTTGAGG
Tph2	GGTTGTCCTTGGATTCTGCTG	GCCTGGATTCGATATGAAGCAT
Maoa	TCGGCAGCCAGTAGGCAGGATT	TTGAGCAGACCAGGCACGGAAG
Slc6a4	TATCCAATGGGTACTCCGCAG	CCGTTCCCCTTGGTGAATCT
5-HT_{1a}	ACAGGCGGCAACGATACTG	AGCACCGCGCAGAAAATGA
5-HT_{1b}	GGGTTCCTCAAGCCAACTTATC	GCCAATAGCATAACCAGCAGT
5-HT_{2a}	TAATGCAATTAGGTGACGACTCG	GCAGGAGAGGTTGGTTCTGTTT
5-HT_{2b}	ACCTGATCCTGACTAACCGTT	TGGGTATTATCACCGCGAGTAT
5-HT_{3a}	CCTGGCTAACTACAAGAAGGGG	TGCAGAAACTCATCAGTCCAGTA
5-HT_{4}	GGCTATATCAATTCGGGGTTGAA	GTGTATGGGCAATTTCTCCAGTT
5-HT_{5a}	ATGGATCTGCCTGTAAACTTGAC	CACTCGGAAAGCTGAGAGAAAA
GAPDH	AGGTCGGTGTGAACGGATTTG	TGTAGACCATGTAGTTGAGGTCA
A. muciniphila	CAGCACGTGAAGGTGGGGAC	CCTTGCGGTTGGCTTCAGAT
A. glycaniphila	TGGGAGCAAGGCGCAAGCT	GCCTTTCACGCCCGACTTAT
A. biwaensis	GCGTCCGCAAGGTGGAAGTG	CGTTGGATTTGGCATAGCCG

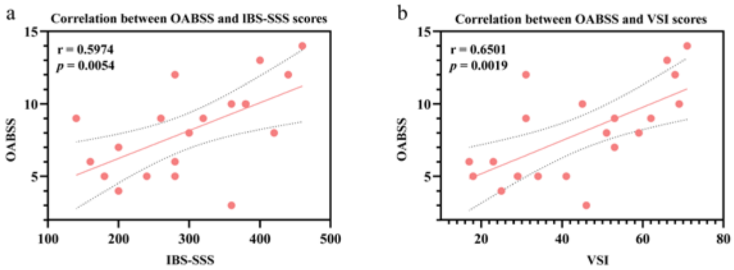

Supplementary Fig. 1 Relationship between bladder, gut, and visceral sensitivity in OAB-IBS patients.

(a) Correlation between OABSS and IBS-SSS scores. (b) Correlation between OABSS and VSI scores. Pearson correlation was used to assess the relationship between the scores.

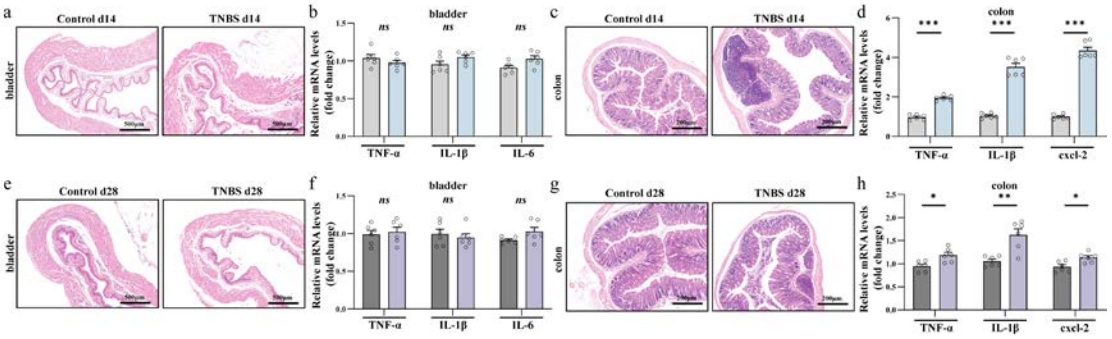

Supplementary Fig. 2 Histological and inflammatory marker analysis of the bladder and colon in the TNBS-induced colitis model.

Histological analysis by H.E staining and gene expression analysis of inflammatory markers by qPCR were performed on bladder and colon tissues collected from control and TNBS-treated mice on day 14 and day 28 post-induction. (a, b) Bladder analysis on day 14. (a) Representative H&E staining of bladder sections. (b) qPCR analysis of inflammatory markers (TNF-α, IL-1β, and IL-6) in the bladder. (c, d) Colon analysis on day 14. (c) Representative H&E staining of colon sections from TNBS-treated mice showing inflammatory cell infiltration. (d) qPCR results showing elevated expression of inflammatory markers (TNF-α, IL-1β, and CXCL-2) in the colon. (e, f) Bladder analysis on day 28. (e) Representative H&E staining of bladder sections. (f) qPCR analysis of inflammatory markers in the bladder. (g, h) Colon analysis on day 28. (g) H&E staining of colon sections showing persistent inflammatory infiltration. (h) qPCR results showing sustained elevation of inflammatory markers in the colon. The statistical difference of various indicators between the two groups was evaluated by a two-tailed unpaired student's T-test and a statistically significant was defined when $p<0.05$. Statistical significance: *$p<0.05$; **$p<0.01$; ***$p<0.001$.

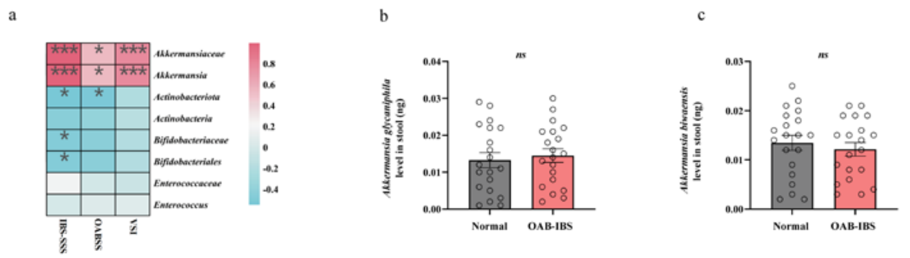

Supplementary Fig. 3 Correlation analysis of gut microbiota with clinical symptoms and species specificity validation. (a) Heatmap of Spearman correlation analysis between the top differentially abundant taxa (identified by LEfSe) and clinical symptom scores (IBS-SSS, OABSS, and VSI). The color scale indicates the correlation coefficient (*r*), with red representing positive correlation and blue representing negative correlation. (b, c) Quantitative PCR analysis of fecal DNA levels for congeneric *Akkermansia* species, including (b) *Akkermansia glycaniphila* and (c) *Akkermansia biwaensis*, in Normal controls and OAB-IBS patients. Data are presented as mean ± SEM. Statistical significance: **p*<0.05; ***p*<0.01; ****p*<0.001.

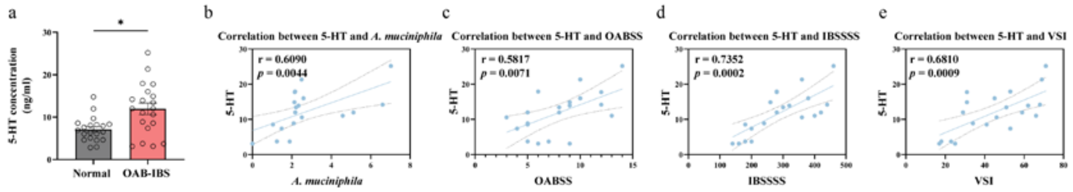

Supplementary Fig. 4 Fecal serotonin levels are elevated in OAB-IBS patients and correlate with *A. muciniphila* abundance and symptom severity.

Fecal samples from healthy controls (Normal, n=20) and OAB-IBS patients (n=20) were analyzed for 5-HT concentration by ELISA and correlated with previously determined *A. muciniphila* abundance (from qPCR) and clinical scores. (a) Boxplot comparing fecal 5-HT concentrations between Normal and OAB-IBS groups. (b) Spearman correlation analysis between fecal 5-HT levels and the abundance of *A. muciniphila*. (c-e) Spearman correlation analyses between fecal 5-HT levels and (c) OABSS, (d) IBS-SSS, and (e) VSI scores in the OAB-IBS patient cohort. The statistical difference of various indicators between the two groups was evaluated by a two-tailed unpaired student's T-test and a statistically significant was defined when *p*<0.05. Pearson correlation was used to assess the relationship between the scores. Statistical significance: **p*<0.05; ***p*<0.01; ****p*<0.001.

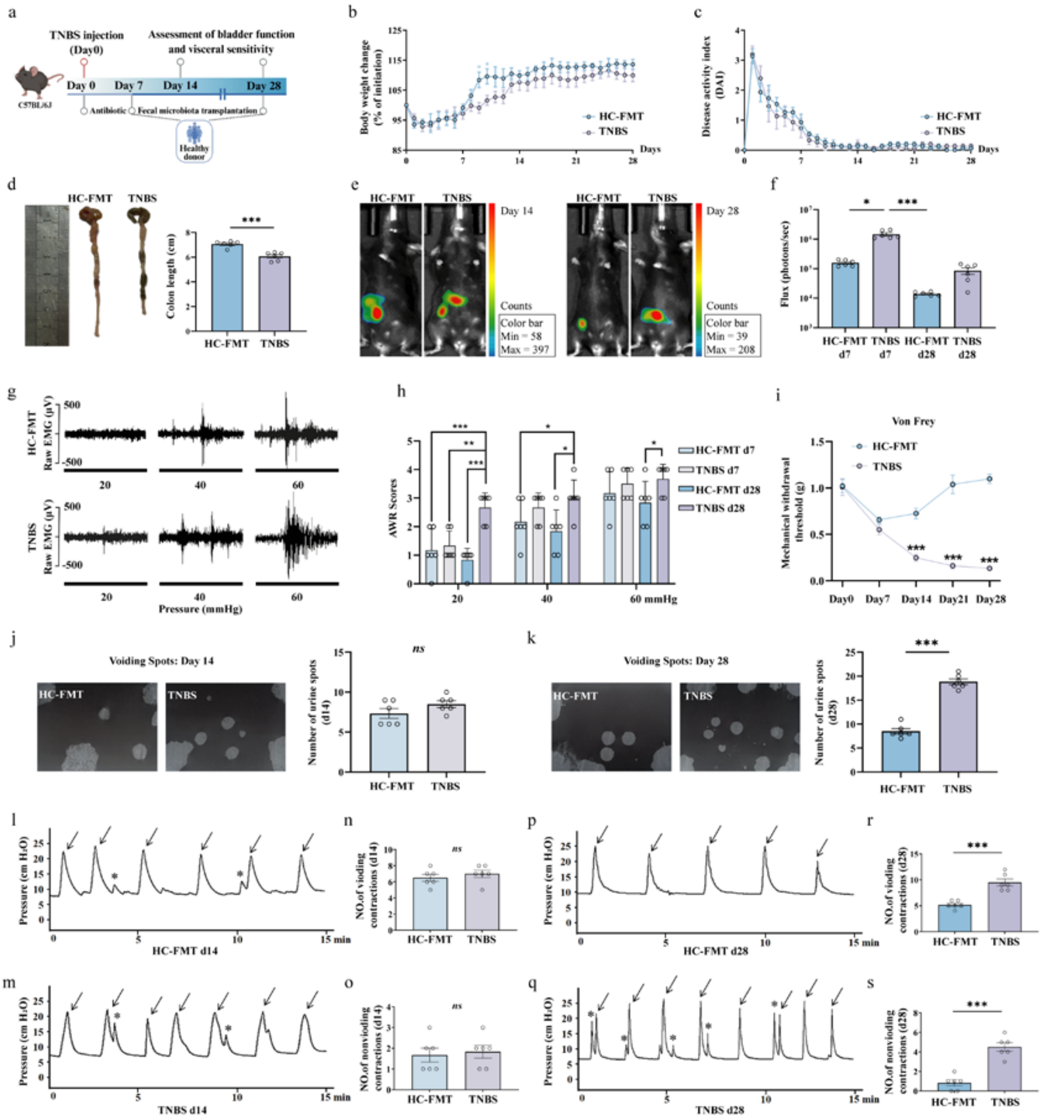

Supplementary Fig. 5 Healthy control microbiota transplantation (HC-FMT) ameliorates TNBS-induced somatic and visceral phenotypes. (a) Schematic diagram of the experimental design. Antibiotic-treated TNBS mice received FMT from healthy donors (HC-FMT). (b) Body weight changes, (c) DAI scores, and (d) colon length. (e) Representative bioluminescence images and (f) quantification of inflammatory signals (Flux) in the abdominal region at days 14 and 28. (g) Representative EMG recordings and (h) AWR scores in response to colorectal distension (CRD) at pressures of 20, 40, and 60 mmHg. (i) von Frey test. (j) Representative voiding spot assay images and quantification of urine spot numbers on day 14. (k) Representative voiding spot assay images and quantification of urine spot numbers on day 28. (l-s) Urodynamic analysis via cystometry. Representative continuous cystometry records on (l-o) day 14 and (p-s) day 28 for both groups. Data are presented as mean ± SEM. The statistical difference of various indicators between the two groups was evaluated by a two-tailed unpaired Student's T-test and a statistically significant was defined when $p<0.05$. Statistical significance: *$p<0.05$; **$p<0.01$; ***$p<0.001$.

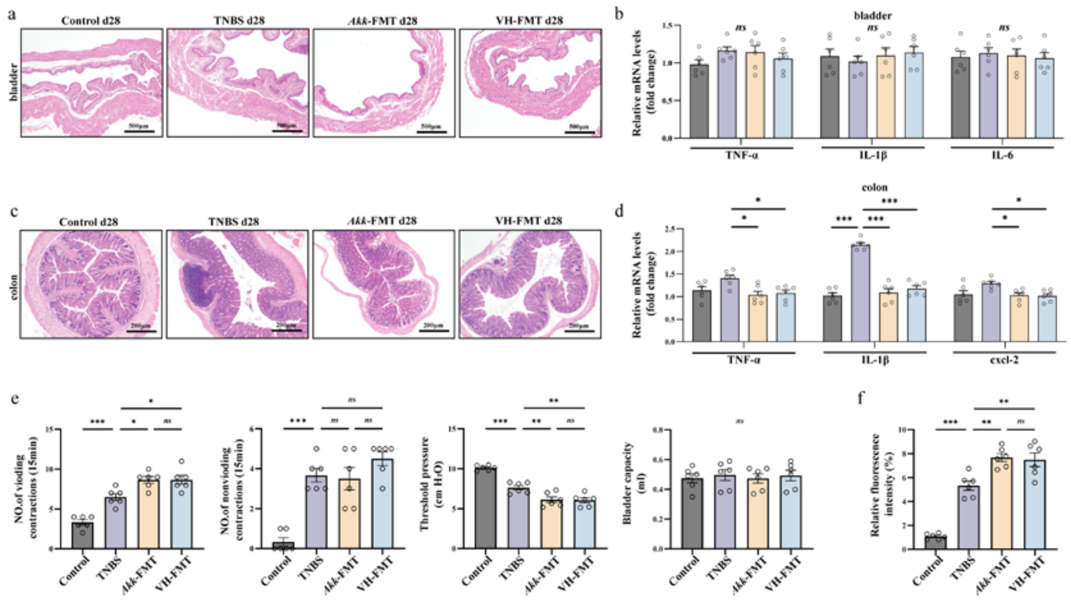

Supplementary Fig. 6 Fecal microbiota transplantation exacerbates bladder dysfunction despite promoting colitis resolution and not inducing cystitis.

Analyses were performed on tissues and data from the four experimental groups described in Fig. 4 (Control, TNBS, *Akk*-FMT, and VH-FMT) on day 28. (a, b) Assessment of bladder pathology. (a) Representative H.E staining of bladder sections and (b) qPCR analysis of inflammatory markers (TNF-α, IL-1β, and IL-6) show no pathological changes in any of the four groups. (c, d) Assessment of colon pathology. (c) Representative H.E staining of colon sections and (d) qPCR analysis of inflammatory markers (TNF-α, IL-1β, and CXCL-2). While the TNBS group still exhibited residual inflammation, both the *Akk*-FMT and VH-FMT groups showed a resolution of inflammation, with marker levels comparable to the control group. (e) The urodynamic parameters evaluated included voiding contractions (arrows), non-voiding contractions (stars), threshold pressure, and bladder capacity. (f) Relative fluorescence intensity of c-fos in DRG sections. Data are presented as mean ± SEM. Statistical differences among groups were evaluated by one-way ANOVA followed by Tukey's multiple comparisons test, and statistical significance was defined when $p<0.05$. Statistical significance: *$p<0.05$; **$p<0.01$; ***$p<0.001$.

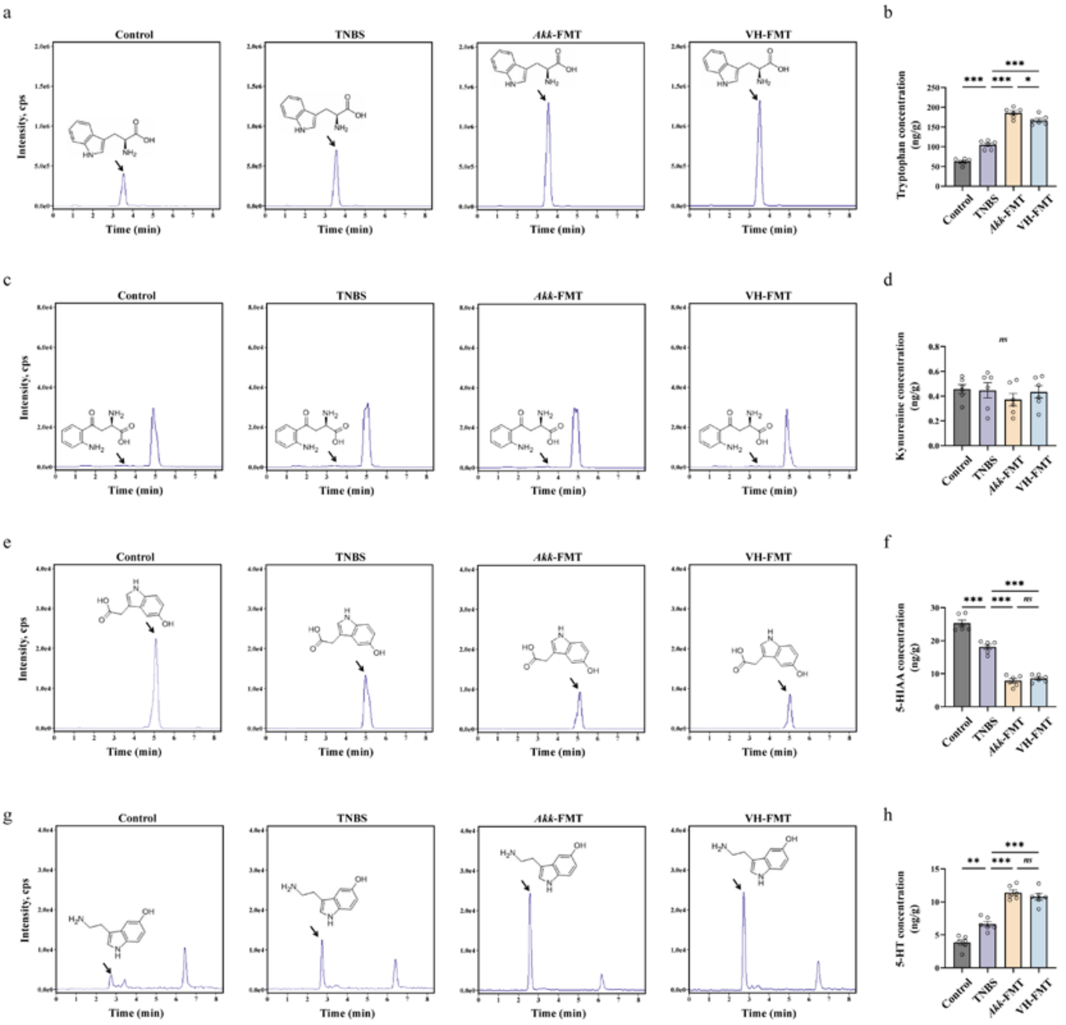

Supplementary Fig. 7 Validation of colonic tryptophan pathway metabolites using targeted LC-MS/MS. To validate the metabolite quantification results obtained by ELISA, targeted liquid chromatography-tandem mass spectrometry (LC-MS/MS) was performed on colonic tissue samples from the Control, TNBS, *Akk*-FMT, and VH-FMT groups. (a, c, e, g) Representative extracted ion chromatograms (XIC) for (a) Tryptophan, (c) Kynurenine, (e) 5-HIAA, and (g) 5-HT. The arrows indicate the specific retention times of the target analytes. (b, d, f, h) Quantitative analysis of colonic concentrations (ng/g tissue) for (b) Tryptophan, (d) Kynurenine, (f) 5-HIAA, and (h) 5-HT. Data are presented as mean ± SEM. Statistical differences among groups were evaluated by one-way ANOVA followed by Tukey’s multiple comparisons test, and statistical significance was defined when $p<0.05$. Statistical significance: *$p<0.05$; **$p<0.01$; ***$p<0.001$.

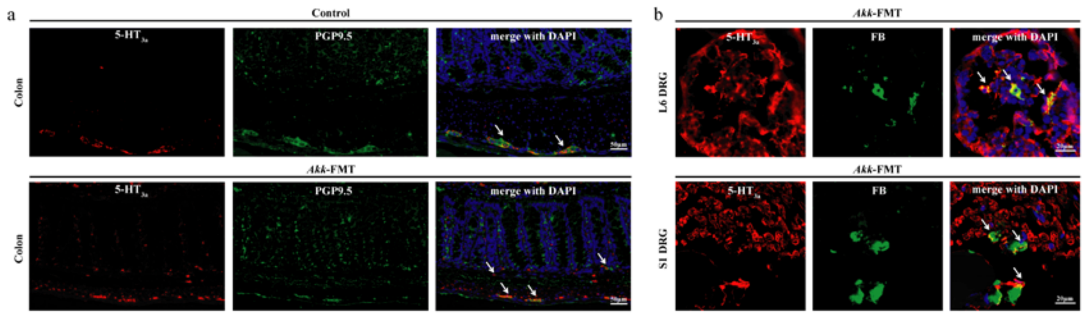

Supplementary Fig. 8 Anatomical localization of 5-HT$_{3a}$ receptors in the colon and dorsal root ganglia.

(a) Representative double immunofluorescence images of colonic sections stained for 5-HT$_{3a}$ receptors (red) and the pan-neuronal marker PGP9.5 (green). Nuclei were counterstained with DAPI (blue). The upper panels show the Control group, and the lower panels show the *Akk*-FMT group. White arrows indicate the co-localization (yellow) of 5-HT$_{3a}$ receptors with PGP9.5-positive nerve fibers in the colonic mucosa. Scale bar = 50 µm. (b) Retrograde tracing combined with immunofluorescence in L6 and S1 DRG from *Akk*-FMT mice. Colon-innervating neurons were labeled by the retrograde tracer Fast Blue (FB, green) injected into the colon wall. Sections were immunostained for 5-HT$_{3a}$ receptors (red). White arrows indicate double-positive neurons (FB+ / 5-HT$_{3a}$+), identifying colon-projecting sensory neurons expressing 5-HT$_{3a}$ receptors. Scale bar = 20 µm.
